# CHAC1 blockade suppresses progression of lung adenocarcinoma by interfering with glucose metabolism via hijacking PKM2 nuclear translocation

**DOI:** 10.1038/s41419-024-07114-6

**Published:** 2024-10-05

**Authors:** Junfan Pan, Sixuan Wu, Qihong Pan, Yuan Zhang, Liu He, Qiwei Yao, Jinyuan Chen, Jiancheng Li, Yiquan Xu

**Affiliations:** 1 Clinical Oncology School of Fujian Medical University, Fujian Cancer Hospital, Fuzhou, China; 2 Department of Radiation Oncology, Fujian Cancer Hospital, Fuzhou, China; 3 The First Affiliated Hospital of Xiamen University, Xiamen, China; 4 School of Basic Medical Sciences, Fujian Medical University, Fuzhou, China; 5 The Central Laboratory, Fujian Key Laboratory of Precision Medicine for Cancer, The First Affiliated Hospital, Fujian Medical University, Fuzhou, China; 6 Department of Thoracic Oncology, Fujian Cancer Hospital, Fuzhou, China

**Keywords:** Cancer metabolism, Tumour biomarkers

## Abstract

Patients with lung adenocarcinoma (LUAD) generally have poor prognosis. Abnormal cellular energy metabolism is a hallmark of LUAD. Glutathione-specific gamma-glutamylcyclotransferase 1 (CHAC1) is a member of the γ-glutamylcyclotransferase family and an unfolded protein response pathway regulatory gene. Its biological function and molecular regulatory mechanism, especially regarding energy metabolism underlying LUAD, remain unclear. By utilizing tissue microarray and data from The Cancer Genome Atlas and Gene Expression Omnibus, we found that CHAC1 expression was markedly higher in LUAD tissues than in non-tumor tissues, and was positively correlated with poor prognosis. Phenotypically, CHAC1 overexpression enhanced the proliferation, migration, invasion, tumor sphere formation, and glycolysis ability of LUAD cells, resulting in tumor growth both in vitro and in vivo. Mechanistically, through a shotgun mass spectrometry-based proteomic approach and high-throughput RNA sequencing, we found that CHAC1 acted as a bridge connecting UBA2 and PKM2, enhancing the SUMOylation of PKM2. The SUMOylated PKM2 then transferred from the cytoplasm to the nucleus, activating the expression of glycolysis-related genes and enhancing the Warburg effect. Lastly, E2F Transcription Factor 1 potently activated CHAC1 transcription by directly binding to the CHAC1 promoter in LUAD cells. The results of this study implied that CHAC1 regulates energy metabolism and promotes glycolysis in LUAD progression.

## Introduction

Lung cancer is a global health concern, with non-small cell lung cancer (NSCLC) accounting for 80–85% of cases [1, 2]. Lung adenocarcinoma (LUAD) is a major subtype of NSCLC. Patients with LUAD have a poor 5-year survival rate of <20%, as many patients are already at an advanced stage of the disease when diagnosed [1]. Additionally, LUAD is characterized by complex mechanisms, hindering the development of effective treatments and technological advancements. In this context, elucidating the molecular mechanisms underlying the development and occurrence of LUAD may facilitate the identification of novel therapeutic targets and strategies.

Notably, few studies have focused on glutathione-specific gamma-glutamylcyclotransferase 1 (CHAC1) [3, 4]. CHAC1 is known as a protein-coding and unfolded protein response (UPR) pathway regulatory gene, but also a candidate gene for glutathione (GSH) catabolism [5]. The function of CHAC1 in tumorigenesis is tumor-type-specific. CHAC1 is upregulated in breast cancer [5, 6] and advanced clear cell renal cell carcinoma (ccRCC) [7] and is associated with poor prognosis. Furthermore, CHAC1 promotes cell proliferation and metastasis in uveal melanoma and is a potential therapeutic target [8]. In contrast, CHAC1 functions as a tumor suppressor gene in primary liver cancer, gastric cancer, prostate cancer [9], and human oral squamous cell carcinoma. The anti-proliferative effect of metformin in gastric cancer may be partly induced by the upregulation of CHAC1 caused via the silencing of lncRNA Loc100506691 [10]. The regulation of CHAC1 and subsequent impact on tumor growth in hepatocellular carcinoma (HCC) remains debated. It has been suggested that CHAC1 is regulated by dihydroartemisinin (DHA), which affects its promoter activity and induces ferroptosis in HCC cells [11]. Alternatively, CHAC1 may be regulated by MIA3, which degrades GSH and leads to HCC progression [12]. However, the expression of CHAC1 at the transcriptional and translational levels and its correlation with the proliferation and metastasis of LUAD remain unclear.

Metabolic alterations have recently received increasing attention in cancer research. Tumor cells exhibit abnormal metabolism, preferentially relying on glycolysis even with adequate oxygen, with elevated rates of glucose uptake and lactate production, rapid production of adenosine triphosphate (ATP) and biomolecules, and accompanied by a reduced rate of oxidative phosphorylation. This metabolic reprogramming phenomenon, known as the Warburg effect or aerobic glycolysis, is a hallmark of cancer cells [13, 14]. In addition to providing cellular energy, aerobic glycolysis can promote uncontrolled proliferation and metastasis of localized tumors [13, 15], regulate the tumor microenvironment, and promote angiogenesis [16] and immune escape [17] to trigger cancer progression. Considering that changes in redox status can affect cellular metabolic homeostasis, CHAC1, as a glutathione metabolism-related enzyme and an upregulated protein in the UPR/endoplasmic reticulum stress process, may influence redox status and regulate cellular metabolism [18]. However, its specific mechanisms remain unclear.

The glycolytic enzyme pyruvate kinase (PK) is a key rate-limiting enzyme that catalyzes pyruvate production. PKM2 contains exon 10 and is mainly present in fetal and tumor tissues, promoting aerobic glycolysis [19]. PKM2 exists in three forms: inactive monomers, less active dimers, and an active tetramer [20]. The tetrameric PKM2 is involved in oxidative phosphorylation and rapidly produces ATP. Dimeric PKM2, in addition to its role as a glycolysis-related enzyme, translocates to the nucleus and acts as a protein kinase and co-transcription factor, activating metabolic and proliferative genes and displaying non-glycolytic functions [20, 21]. However, the dynamic changes in PKM2 during tumorigenesis and the molecular mechanism of nuclear localization of PKM2 are not fully understood.

Here, we demonstrated that CHAC1 promotes the nuclear translocation of PKM2 by binding to ubiquitin-like modifier activating enzyme 2 (UBA2) to activate the small ubiquitin-related modifier modification (SUMOylation) of PKM2, which in turn activates glycolytic gene expression and promotes LUAD progression. Our study highlighted the significance of CHAC1 in LUAD progression and provides new insights into carcinogenesis and potential targets for cancer therapy.

## Materials and methods

### Patients and specimens

A total of 30 clinical samples (cohort 1) were collected from the Fujian Cancer Hospital between January 1, 2018, and October 1, 2022. Matched tumor and adjacent normal tissue samples were collected from the 30 enrolled patients; these were cut into 1 cm^3^ pieces within 30 min of harvesting, frozen overnight in liquid nitrogen, and stored at −80 °C for quantitative real-time PCR and western blot detection. LUAD was diagnosed via histopathology. The patients did not receive radiotherapy, chemotherapy, targeted therapy, or any other biological therapy before surgery.

The tumor node metastasis (TNM) stage of LUAD was defined according to the 7th edition of the American Joint Committee on the Cancer TNM staging system. Informed consent was obtained from each patient before conducting the study. This study was approved by the Ethics Committee of Fujian Cancer Hospital (K2023-139-01).

A tissue microarray (TMA) (cohort 2) composed of LUAD specimens (*n* = 98) and adjacent normal tissue specimens (*n* = 82), containing detailed clinicopathological information from 81 cases, was purchased from Outdo Biotech Ltd. (HLugA180Su08, Shanghai, China) for immunohistochemical (IHC) staining. Based on the average CHAC1 IHC score (mean value = 5.93), the patients were divided into high (*n* = 46) and low (*n* = 35) CHAC1 expression groups for clinical analysis. Ethical approval for the TMA study was granted by Outdo Biotech’s Clinical Research Ethics Committee (SHYJS- CP-1904014).

### Analysis of public clinical datasets

The fragments per kilobase of transcript per million mapped reads of the LUAD transcriptome data, and the clinical information for 535 LUAD samples and 59 adjacent normal tissues, were obtained from The Cancer Genome Atlas (TCGA) database (https://portal.gdc.cancer.gov/), including 58 paired samples. ROC analysis was performed using the pROC (1.18.0) package, and the results were visualized using the ggplot2 package in R. Pathway-enrichment analysis was performed using Gene-Set-Enrichment Analysis (GSEA) 2.0.9 software (http://www.broadinstitute.org/gsea/). The NCBI Gene Expression Omnibus datasets GSE31210 and GSE1007 were used to analyze CHAC1 expression in cancer and adjacent normal tissues. The GSE31210 dataset was used for patient overall survival (OS) analysis (http://www.ncbi.nlm.nih.gov/geo/). The University of ALabama at Birmingham CANcer data analysis Portal (UALCAN) database [22, 23] was used to perform correlations between CHAC1 protein expression levels and clinical factors, such as the TNM and pathological stage, and OS analysis.

### Cell transfection

The human CHAC1 overexpression lentivirus was constructed using the pLVZ vector (Zolgene, Fuzhou, China). Lentiviral shRNAs targeting CHAC1 were constructed using GV344 and pLV vectors (GeneChem, Shanghai, China), lentiviral shRNAs targeting PKM2 were constructed using GV344 and pLV vectors (Hanbio, Shanghai, China), respectively. To stabilize transfection, LUAD cells were seeded in 6-well plates at ~60% confluence overnight and then infected with the corresponding lentivirus in the presence of polybrene (4 µg/ml). Twenty-four hours after transfection, the medium was replaced with fresh medium. Transfected cells were selected with puromycin (5 µg/ml). siRNAs targeting E2F1, MAZ, and PKM2 were synthesized by HippoBio Technology (Huzhou, China). siRNAs targeting UBA2 were synthesized by Hanbio. The HA-tagged PKM2 wild-type plasmid and the His-tagged PKM2^I267/268A^ plasmid were cloned into the GV658 vector (GeneChem). The Flag-tagged SUMO1 plasmid was cloned into the CMV vector (Hanbio). The 2000-bp sequence upstream of the CHAC1 promoter was inserted into the pgl4.10 vector to generate a luciferase reporter plasmid. Transient transfection was performed using Lipofectamine 3000 (Invitrogen, Carlsbad, CA, USA) according to the manufacturer’s protocol. Cells were harvested 48–72 h after transfection. The sequences of shRNAs and siRNAs used in this study are listed in Supplementary Table S1.

### Immunohistochemical (IHC) staining

IHC staining was performed for tissue sections, TMA, and xenograft tumors. Briefly, sections were incubated with primary antibodies to CHAC1, PKM2, and Ki67 overnight at 4 °C. Normal goat serum was used as a negative control. Sections were washed and incubated with a biotinylated anti-rabbit secondary antibody (Roche, Switzerland), followed by streptavidin-horseradish peroxidase complex (Dako, Carpinteria, CA, USA). The slices were soaked in 3,3-diaminobenzidine, counterstained with 10% Mayer’s hematoxylin, dehydrated, and mounted. TMA images were captured at 40× magnification using an Aperio GT450 scanner (Leica Biosystems). Sliced xenograft tumor images were captured at 100× magnification using the same scanner. The staining intensity was scored as follows: no intensity, 0; weak intensity, 1+; moderate intensity, 2+; and strong intensity, 3+. The staining extent score, based on the percentage of positively stained cells, was categorized as 0 (0%), 1 (1–25%), 2 (26–50%), 3 (50–75%), or 4 (76–100%). The final histological score (H-score) was calculated by multiplying the staining intensity score with the staining extent score. These scores were determined independently by two senior pathologists who were blinded to the clinical and pathological information. The primary antibodies used are listed in Supplementary Table S2.

### Tumor sphere formation

Tumor spheroid formation was performed as previously described [24]. Cells (4 × 10^4^) were seeded in 6-well ultra-low adsorption plates and then cultured in a saturated humidity atmosphere of 5% CO_2_ at 37 °C to form tumor spheres. After 2 weeks, images of the cells were scanned using an inverted microscope at 200× magnification. The number of tumor spheres was counted and plotted, and the percentage of tumor spheres >100 µm in diameter was calculated using Image J software.

### Wound-healing assay

For in vitro wound-healing assays, transfected A549 and PC9 cells were seeded into 6-well plates. The indicated cells were grown to confluency as monolayers and then scratched with a 10-µl pipette tip. The cells were washed with PBS to remove floating cells, and the scratch was imaged and recorded at 0 h. The cells were then incubated in 1% serum medium for 48 h, imaged again, and the percentage of the scratched area covered with cells was determined using Image J software.

### Migration and invasion assays

Migration and invasion assays were performed using transwell chambers with 8.0-μM pore sizes (Corning, USA). For the migration assays, 1 × 10^5^ transfected cells were resuspended in 200 µl serum-free 1640 medium and added to the upper chambers, while 2 × 10^5^ transfected cells in chambers coated with Matrigel were used for the invasion assay. For both assays, the bottom chambers were filled with RPMI 1640 medium containing 10% FBS. After 24 h, the migrating and invasive cells on the membranes were stained with 0.5% crystal violet and photographed under a microscope, and the cell numbers were counted using Image J software.

### Flow cytometry (FACS) for cell-cycle analyses

Cell-cycle analysis was performed using the Cell-Cycle and Apoptosis Analysis Kit (Meilunbio, China, MA0334). A total of 1 × 10^6^ cells were collected, and 1 ml PBS was added to induce the resuspension of single cells. Precooled absolute ethanol (3 ml) was slowly added, and the mixture was allowed to stand overnight at 4 °C, after which it was centrifuged at 1000 × *g* for 3–5 min. The mixture absorbed the supernatant, and 1 ml PBS was again added to cause resuspension. Staining buffer: PI (20×): RNase A (50×) = 100:5:2 was used to prepare the working solution for staining. Staining working solution (500 μl) was added to each tube of the cell sample, and red fluorescence was detected at an excitation wavelength of 488 nm via flow cytometry after a warm bath at 37 °C in the dark for 30 min.

### Immunoprecipitation (IP) analysis

The cells were lysed with RIPA lysis buffer (Beyotime, Shanghai, China) and supplemented with a protease inhibitor cocktail (Beyotime). Cell lysates were incubated with specific primary antibodies and protein A/G magnetic beads (Smart-Lifesciences, China, SM015005), and normal IgG served as the control. The magnetic beads were washed thrice with TBS buffer (50 mM Tris-HCl and 150 mM NaCl, pH 7.4), boiled, and analyzed by using western blotting.

### Silver staining and shotgun mass spectrometry (MS)

IP was performed as previously described. Bound proteins were separated using SDS–PAGE (LABLEAD, Beijing, China) and stained using a Silver Staining Kit (Beyotime, Shanghai, China). The protein solution or strips were digested into peptide mixtures via protease and then separated by high-performance liquid chromatography (HPLC), followed by serial transfer into an Orrap Discovery mass spectrometer (Thermo) for LC–MS/MS analysis. After the peptides were ionized in the mass spectrometer, the mass-charge ratio (m/z) of each peptide was obtained by detector analysis, and a mass spectrometer further bombarded the peptide ions to obtain the secondary mass spectrum signal. Mascot2.6, Proteome Discoverer2.2, NCBInr, and UniProt were used for protein identification.

### High-throughput RNA sequencing

Total RNA was extracted using the TRIzol reagent (Invitrogen) according to the manufacturer’s instructions. RNA integrity and concentration were assessed using the RNA Nano 2100 assay kit (Agilent Technologies, USA) and Bioanalyzer 6000 system. Total RNA was >2 μg/sample. Libraries were constructed using Illumina’s NEBNext® UltraTM RNA Library Prep Kit. The libraries were quantified and detected using a Qubit2.0 Fluorometer with an Agilent 2100 bioanalyzer. Illumina sequencing was performed after a qualified library inspection. The sequencing sequences were converted into sequence data (reads) using CASAVA base recognition, and the files were in FASTQ format. The HISAT tool was used to align sequences to the reference genome. StringTie (1.3.3b) [25] was used for novel gene prediction. FeatureCounts (1.5.0-p3) were used to calculate the reads mapped to each gene, and DESeq2 software (version 1.16.1) was used for differential expression scores. The R package “edgeR” was utilized to explore the differentially expressed genes with the fold change criteria >2 and *p* value < 0.05. ClusterProfiler (version 3.4.4) software was used to perform GO and KEGG pathway-enrichment analyses.

### Immunofluorescence staining

The cells were seeded in a 24-well chamber slide. The next day, the cells were fixed with 4% formaldehyde, permeabilized with 0.3% Trion X-100, blocked with QuickBlock™ blocking solution (Beyotime, China), and incubated with the specific antibodies overnight. After washing with PBS, the cells were incubated with Alexa Fluor 488 and/or Alexa Fluor 594 (1:1000 dilution; Abcam, UK) for 2 h. The nuclei were stained with DAPI, and the cells were imaged using a laser confocal microscope (NIKON Eclipse Ti, Japan). The primary antibodies used are listed in Supplementary Table S2.

### Chromatin immunoprecipitation (ChIP) assays

A ChIP kit (Thermo Fisher Scientific, USA) was used according to the manufacturer’s protocol. Briefly, cells (1 × 10^7^ cells/condition) were crosslinked, lysed, and sonicated to obtain DNA samples. DNA fragments were immunoprecipitated with an E2F1 antibody (Proteintech, China), and the binding of E2F1 to the CHAC1 promoter region was analyzed via RT-qPCR using specific primers for ChIP-enriched DNA fragments and normalized to control IgG (Supplementary Table S1).

### Dual-luciferase reporter assay

Cells were seeded in 96-well plates and co-transfected with plasmids containing the E2F1 and MAZ sequences cloned into pCMV, pGL4.10 luciferase reporter vector containing the CHAC1 promoter (Promega, USA), and pRL-TK. Lysates were prepared 48 h after transfection, and the luciferase activity was measured using a dual-luciferase reporter assay system (Promega, USA) according to the manufacturer’s protocol. The luciferase activity was calculated as the ratio of firefly to Renilla luciferase activity.

### Glycolysis analysis

Assay kits for glucose uptake colorimetry, lactate colorimetry, ATP, hexokinase (HK), fructose phosphokinase (PFKP), PK, and LDH activity (Jiancheng Corporation, Nanjing, China) were used according to the manufacturer’s instructions.

For the glucose-uptake experiment and lactic acid colorimetric assay experiment, cells were collected at a density of more than 1 × 10^6^ cells/ml and washed with isotonic buffer (0.1 mol/L, pH 7–7.4 phosphate buffer). The supernatant was discarded, and the cell precipitate was retained. Following this, 0.2–0.3 ml of homogenization medium (0.1 mol/L, pH 7–7.4 phosphate buffer) was added for homogenization. The prepared homogenate was added to a 96-well plate, and the absorbance at 505 nm was measured using a microplate reader. In the ATP assay experiment, 1 × 10^6^ cells were collected, 300–500 µl hot double-distilled water was added, and the cells were homogenized and broken in a hot water bath (90–100 °C). The water bath was boiled for 10 min. The absorbance at a wavelength of 636 nm was measured by using a microplate reader. In the HK kit, fructose PFKP-activity assay, PK test and LDH activity test experiments, 5 × 10^6^ cells were collected and added to 1 ml of the prepared reagent and lysed via ultrasound, the supernatant was collected, and the absorbance was determined using a microplate reader.

### Seahorse-XF24-extracellular-acidification rate (ECAR) and oxygen consumption rate (OCR)

ECAR and OCAR measurements were performed using a hippocampal XF24 extracellular flux analyzer (Hippocampal, USA). After trypsinization of the cells, cancer cells were seeded in hippocampal XF24 cell plates (1 × 10^4^/well) and cultured for 24 h in a humidified 37 °C, 5% CO_2_ incubator. The cell plates were then incubated at 37 °C in a 0% CO_2_ incubator for 60 min. For ECAR measurements, the Seahorse XF Glycolytic Stress Test Kit was used, with the medium replaced by Seahorse XF Base Medium sequentially supplemented with glucose (10 mM), oligomycin (1 μM and 2 μM), and 2-deoxyglucose (2-DG, 80 mM) according to the manufacturer’s protocol. OCR was measured using the Seahorse XF cell mito stress test kit, with the medium replaced by Seahorse XF base medium sequentially supplemented with oligomycin (1.5 μM), FCCP (0.5 μM), and rotenone (0.5 μM) following the manufacturer’s instructions.

### Mouse xenograft study

The animal experimental protocol involved in this study was reviewed and approved by the Animal Ethics Committee of Fujian Medical University (No. IACUC FJMU 2023-0109). BALB/c nude mice (4–6-week-old) were obtained from GemPharmatech Co., Ltd., Jiangsu, China. All mice were fed a standard rodent diet and maintained under specific pathogen-free conditions with an ambient temperature of 23 °C and a 12-h/12-h light/dark cycle. Nude mice were randomly assigned to groups and injected with 5 × 10^6^/100 µl A549 cells transfected with overexpression lentivirus (*n* = 6/group), PC9 cells transfected with knockdown lentivirus (*n* = 6/group), and A549 cells transfected with CHAC1 overexpression lentivirus combined with PKM2-knockdown lentivirus (*n* = 8/group) to establish a subcutaneous xenograft model. The longest (*A*) and shortest (*B*) diameters of the grafts were measured weekly, and the tumor volume was monitored for 4 weeks after inoculation (calculated as 0.5 × *A* × *B*^2^). After 4 weeks, or when the tumors reached a diameter of 1.5 cm, the mice were euthanized, and the subcutaneous tumors were harvested. The specimens were weighed and subjected to IHC staining.

### Statistical analysis

Statistical analyses were performed using GraphPad Prism 7.0. All the data are presented as the mean ± SD and represent at least three independent experiments. Spearman’s rank correlation test was used to analyze the correlation between gene expression in tissue samples. ANOVA was used to compare the means between the treatment groups. Student’s *t*-test was used for unpaired observations, with *p* < 0.05 being considered significant. Correlation analyses were performed using the *R*-value test. OS was defined as the time from surgery to death from LUAD or the last follow-up. The Kaplan–Meier method and log-rank test were used for survival analysis. All the tests were two-tailed.

## Results

### Upregulation of CHAC1 correlates with aggressive phenotype and poor prognosis in LUAD

**Fig. 1:**
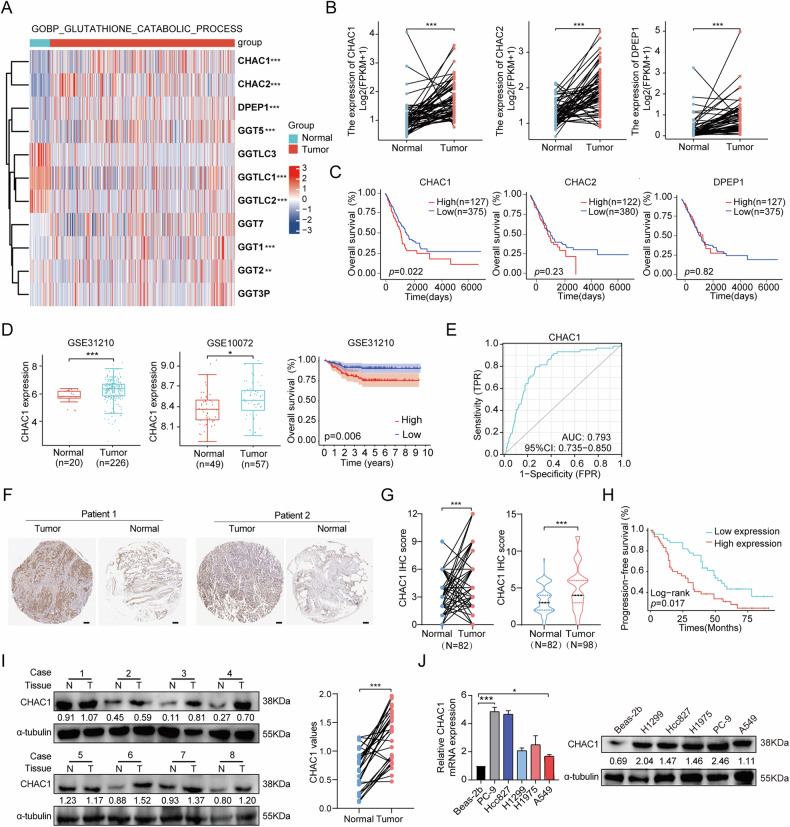
High CHAC1 expression was significantly associated with poor prognosis in LUAD. **A** Transcription levels of GSH catabolic genes in LUAD tissues (*n* = 535) and adjacent normal tissues (*n* = 59) from TCGA database (screened by logFC > 1, *p* < 0.05). **B** Transcription levels of DPEP1, CHAC2, and CHAC1 in 58 matched tumor and adjacent normal tissue samples of LUAD in TCGA database. **C** Kaplan–Meier curves for OS based on high and low DPEP1, CHAC2, and CHAC1 levels in TCGA database. **D** GEO datasets (GSE31210, GSE10072) were used to analyze CHAC1 expression and OS in LUAD. **E** Diagnostic ROC was used to analyze CHAC1 expression in predicting LUAD accuracy. **F**, **G** IHC staining for CHAC1 in LUAD tissue microarray. **H** Kaplan–Meier curves for OS according to high and low CHAC1 levels of LUAD tissues in cohort 2. **I** Protein levels of CHAC1 were detected in primary LUAD tissues (T) and corresponding adjacent nontumoral lung (N) tissues. **J** mRNA and protein expression levels of CHAC1 in Beas-2b and LUAD cell lines were detected by using RT-qPCR and western blot. Beas-2b was used as internal control. Three independent experiments were performed, and data are means ± SD from one representative experiment (*n* = 3). Values below western blot bands are optical density of signal. *p < 0.05, **p < 0.01, ***p < 0.001.

To identify the potential clinical significance of GSH-catabolism genes in human LUAD, we first analyzed the mRNA expression levels of 11 GSH-catabolism genes in primary tumors and adjacent normal tissues using the TCGA dataset. The three most highly expressed genes were DPEP1 (log_2_FC = 1.61, *p* < 0.001), CHAC2 (log_2_FC = 1.20, *p* < 0.001), and CHAC1 (log_2_FC = 1.04, *p* < 0.001) (Fig. 1A and Supplementary Table S3). In the 58 matched samples, the expression of CHAC1, CHAC2, and DPEP1 was significantly higher in LUAD tissues than in the adjacent normal lung tissues (*p* < 0.001) (Fig. 1B). Notably, patients with LUAD and high CHAC1 expression had worse OS than those with low CHAC1 expression (*p* < 0.05). However, CHAC2 and DPEP1 expression did not affect LUAD OS (Fig. 1C). In addition, the results of CHAC1 in the TCGA dataset were verified in the GEO datasets, including GSE31210 and GSE10072 (Fig. 1D). Moreover, high CHAC1 expression was associated with advanced TNM stage and N stage (*p* < 0.05) (Supplementary Fig. 1). We also found that CHAC1 expression was highly accuracy in predicting LUAD (AUC = 0.793, Fig. 1E).

Subsequently, we used a TMA containing 98 LUAD tissues and adjacent normal lung tissues to further evaluate the expression of CHAC1 in LUAD. IHC staining showed that CHAC1 was mainly distributed in the cytoplasm and cell membrane of LUAD cells, and its expression was higher than that in normal lung tissues (Fig. 1F, G). These results are consistent with those of the public database analysis. Survival analysis revealed that patients with LUAD with high CHAC1 expression had significantly worse OS than those with low CHAC1 expression (*p* = 0.017, Fig. 1H). Moreover, in 81 samples with clinical information, CHAC1 expression was significantly associated with a worse T stage (Supplementary Table S4) and was an independent prognostic factor for poor prognosis in patients with LUAD (Supplementary Table S5).

Next, the CHAC1 mRNA and protein expression levels in vitro were assessed. Thirty fresh tissue specimens were collected, and CHAC1 was significantly more highly expressed in cancerous tissues than in paracancerous tissues (*p* < 0.001, Fig. 1I). Furthermore, the expression of CHAC1 in the LUAD cell lines was higher than that in normal lung epithelial cells (Beas-2b) (Fig. 1J). These results indicate a positive correlation between CHAC1 expression and the aggressive progression and poor prognosis of LUAD.

### CHAC1 promotes LUAD proliferation and metastasis

**Fig. 2:**
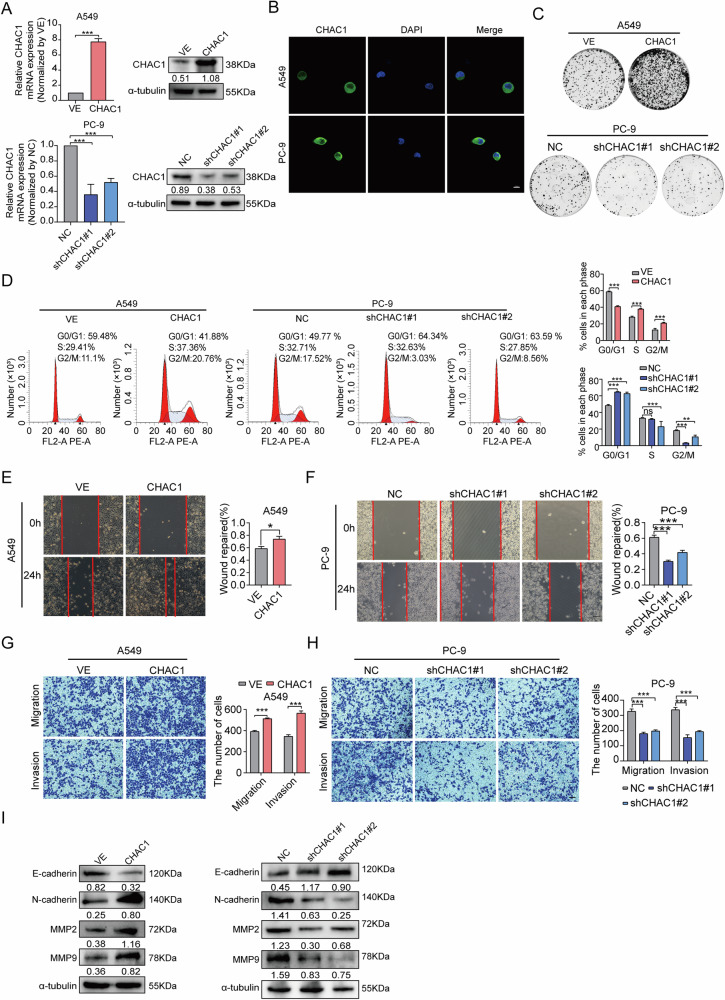
CHAC1 promotes proliferation and metastasis of LUAD cells. **A** CHAC1 overexpression in A549 and shCHAC1 PC9 cells was constructed via transfecting lentivirus with overexpression of shRNA and verified by using western blotting and qRT-PCR. B-actin was used as a loading control. **B** IF staining was used to detect the distribution of CHAC1 in cells. Scale bars, 10 µm. **C** Colony-formation assay, performed to detect the effect of overexpression or knockdown of CHAC1 on cell proliferation. **D** Flow cytometry was used to analyze the effect of overexpression or knockdown of CHAC1 on cell-cycle progression. **E**–**H** Wound-healing assay and transwell assay were used to detect cell migration and invasion abilities after overexpression or knockdown of CHAC1. Scale bars, 200 µm. **I** Western blotting was used to detect the effect of overexpression or knockdown of CHAC1 on EMT. Results are shown as means ± SD. Values below western blot bands are optical density of signal. *p < 0.05, **p < 0.01, ***p < 0.001, ns no statistical significance.

To explore the functional role of CHAC1 in LUAD, we constructed a CHAC1-FLAG structure by fusing the tag with a full-length CHAC1 transcript. Stable CHAC1-overexpressing cell lines (OE) were generated from the A549 cell line, which exhibits relatively low CHAC1 expression, with Vector used as the positive control (VE). CHAC1-knockdown cell lines (sh1, sh2, and sh3-CHAC1) were established from the PC9 cell line, which has relatively high CHAC1 expression, and a negative control (NC) was prepared. The overexpression and knockdown efficiency of CHAC1 in these cell sublines was detected via western blotting and RT-qPCR; sh2- and sh3-CHAC1 showed greater inhibition of CHAC1 expression (Fig. 2A and Supplementary Fig. 2A) and were renamed shCHAC1 #1 and shCHAC1 #2 for subsequent experiments. As shown in Fig. 2B, CHAC1 was mainly distributed in the cell membrane and cytoplasm.

Based on the clone-formation assay, CHAC1 overexpression significantly promoted LUAD cell proliferation, whereas CHAC1 depletion markedly decreased the proliferative capacity of LUAD cells (Fig. 2C and Supplementary Fig. 2B, C). Pathway-enrichment analysis showed that the cell cycle G1_S phase transition (*p* < 0.05) and G2_M transition (*p* < 0.05) were significantly positively correlated with the expression level of CHAC1 in the TCGA dataset (Supplementary Fig. 2D). Thus, we further performed cell-cycle analysis and found that the proportion of cells in the S and G2/M phases increased in CHAC1-overexpressing A549 cells, whereas the CHAC1-knockdown group exhibited a larger proportion of cells in the G0/G1 phase (Fig. 2D). These results suggest that CHAC1 is an important regulator of cell proliferation in LUAD.

In addition, the migration and invasion capacities of LUAD cells were significantly enhanced when CHAC1 was overexpressed, with opposite trends being observed under CHAC1 depletion (Fig. 2E–H and Supplementary Fig. 2E, F). Since the epithelial–mesenchymal transition (EMT) process is integral to tumor metastasis [26], we further investigated the relationship between CHAC1 expression and EMT. As shown in Fig. 2I, CHAC1 overexpression inhibited E-cadherin expression and enhanced the expression levels of N-cadherin, MMP2, and MMP9, while the knockdown of CHAC1 had the opposite effect. We also detected the effect of CHAC1 expression on the tumor sphere-forming capacity. Compared to the control group, the overexpression of CHAC1 in A549 cells increased the size and number of tumor spheres. Conversely, the depletion of CHAC1 in PC9 cells reduced the size and number of tumor spheres (Supplementary Fig. 2G). Collectively, CHAC1 expression contributes to the proliferation and metastasis of LUAD cells.

### CHAC1 enhances glycolysis of LUAD cells

**Fig. 3:**
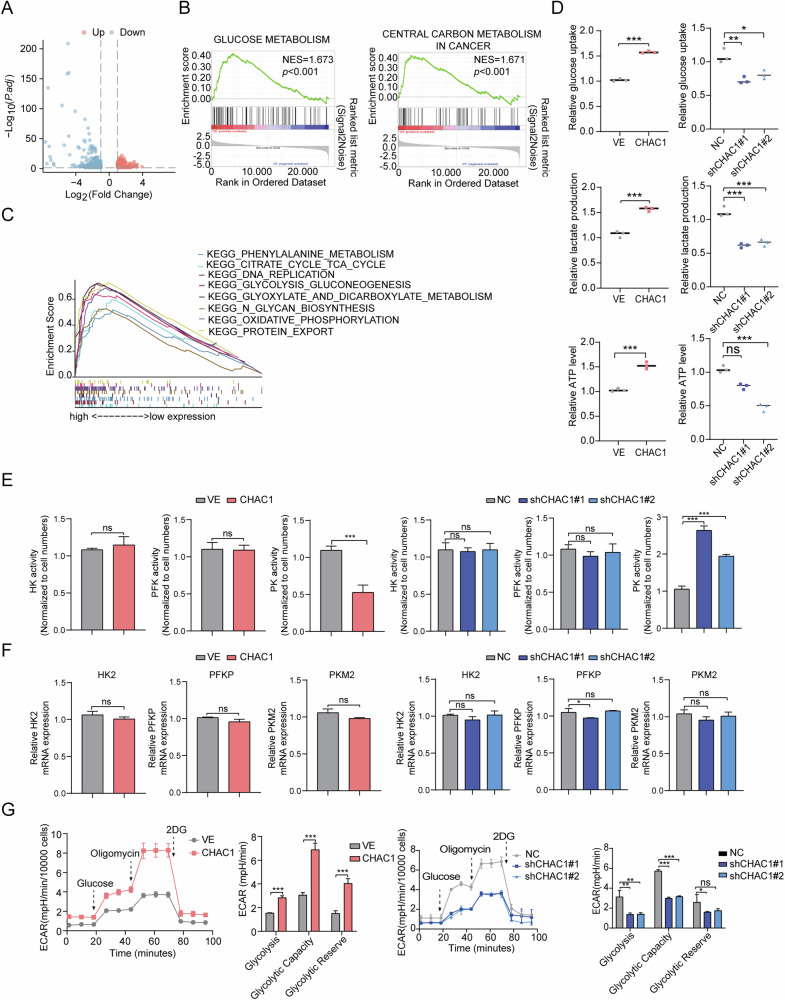
CHAC1 is essential for the glycolytic activity of LUAD. **A** Volcano plots depict upregulated (red) and downregulated (blue) genes in CHAC1 OE cells. **B** Differential genes in CHAC1 OE cells were subjected to GSEA. **C** The pathways involved in CHAC1 expression were analyzed based on TCGA database. **D** Glucose uptake, lactate production, and ATP levels in CHAC1, OE, and knockdown cells were quantified. **E** Enzymatic activity reactions of HK, PFK, and PK in CHAC1, OE, and knockdown cells were detected. **F** Relative HK, PFKP, and PKM2 mRNA levels in CHAC1, OE and knockdown cells were analyzed. **G** ECAR of overexpression and knockdown of CHAC1 were monitored, and glycolysis, glycolytic capacity, and glycolytic reverse were calculated. Results are shown as means ± SD. NES normalized enrichment score, FDR false discovery rate. *p < 0.05, **p < 0.01, ***p < 0.001, ns no statistical significance.

To investigate the molecular mechanism of CHAC1 in LUAD, we performed an unbiased transcriptomic analysis using RNA-seq in A549 cells with or without overexpression of CHAC1. The results showed that the expression levels of 768 genes were affected by CHAC1 overexpression, of which 310 genes were upregulated and 458 genes were downregulated (Fig. 3A). The KEGG and GO analyses revealed that the pathways were enriched in these differentially expressed genes (Supplementary Fig. 3A). The GSEA revealed that the target genes were significantly involved in the energy-metabolism signaling pathway (Fig. 3B). GSEA was also performed on the mRNA expression profile of LUAD in the TCGA dataset, and showed that the activity of the energy metabolism-related pathways, including glycolysis/gluconeogenesis and the citrate cycle (TCA cycle), was positively correlated with CHAC1 expression (NES > 1.5, *p* < 0.05, Fig. 3C).

Next, we examined the association between CHAC1 expression and glycolytic phenotype. The upregulation of CHAC1 enhanced glucose uptake, lactate production, and ATP levels, whereas the downregulation of CHAC1 showed the opposite results (Fig. 3D). Since the glycolytic pathway is controlled via several key glycolytic rate-limiting enzymes, including HK, PFK, and PK [27], we aimed to determine whether CHAC1 expression affects key glycolytic rate-limiting enzymes. The results showed that the downregulation of CHAC1 enhanced PK activity, while the knockdown of CHAC1 had the opposite effect; however, the activities of HK and PFK were not affected by the expression of CHAC1 (Fig. 3E). Interestingly, we detected the glycolytic rate-determining enzymes HK2, PFKP, and PKM2 via quantitative PCR. The results showed that interfering with the expression of CHAC1 did not affect the mRNA levels of HK2, PFKP, or PKM2 (Fig. 3F). Moreover, we detected the impact of CHAC1 expression on ECAR, an indicator of overall glycolytic flux and OCAR, an indicator of mitochondrial respiration. The upregulation of CHAC1 enhances glycolysis and glycolytic capacity in LUAD cells, evidenced by a significant increase in ECAR. Conversely, CHAC1 knockdown produces the opposite effect. Notably, alterations in CHAC1 expression do not significantly impact OCAR in LUAD cells (Fig. 3G and Supplementary Fig. 3B). These results indicated that CHAC1 may control glycolytic metabolism by specifically regulating PK activity.

CHAC1 is also known to act as a γ-glutamylcyclotransferase, leading to GSH depletion and possibly inducing ferroptosis [25]. Therefore, we examined whether altering CHAC1 expression in LUAD cells would result in GSH depletion and ferroptosis. Results showed that CHAC1 overexpression reduced the total intracellular GSH and induced intracellular lipid peroxidation, while CHAC1 deficiency had the opposite effect (Supplementary Fig. 3C, D). However, we examined the proportion of 7-AAD-positive cells and found that alterations in CHAC1 expression did not significantly affect cell death (Supplementary Fig. 3E). Moreover, the additional use of ferroptosis inhibitor (ferrostatin-1, Fer-1) could not abolish the enhanced cellular glycolytic capacity caused by CHAC1 overexpression (Supplementary Fig. 3F), which mean that the enhancement of glycolytic pathway is not dependent through the glutathione depletion role of CHAC1.

### CHAC1 interacts with PKM2 and prevents its degradation

**Fig. 4:**
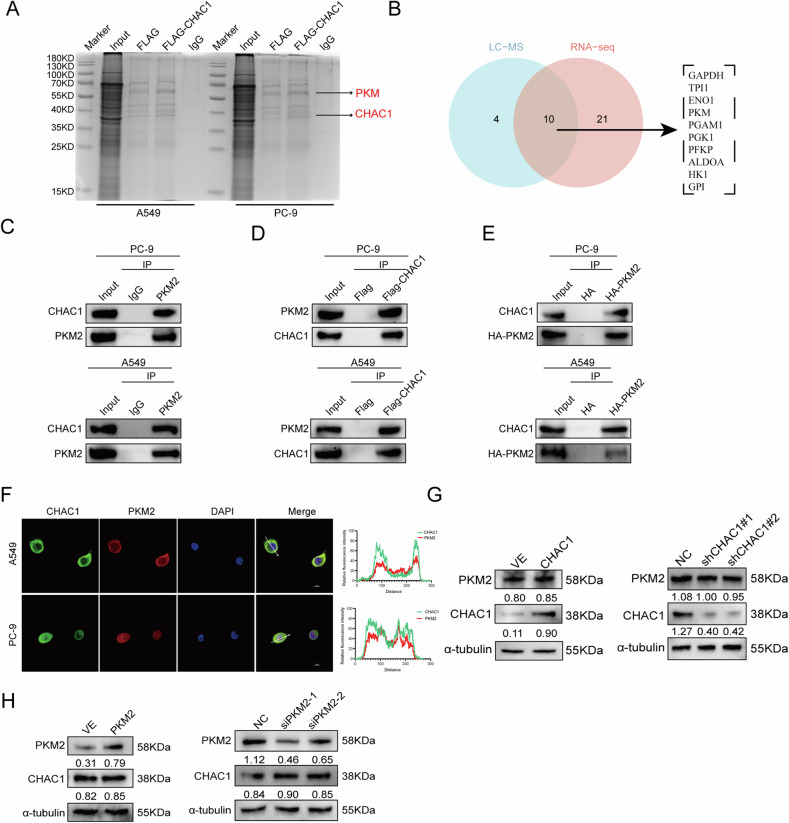
CHAC1 interacts with and stabilizes PKM2. **A** Identification of CHAC1-interacting proteins by using silver staining. Indicated bands were sequenced via mass spectrometry analysis. **B** Proteins in LC–MS “glycolytic process” pathway intersect with those in “glucose metabolism” pathway in RNA-seq. **C** Endogenous interaction between CHAC1 and PKM2 in LUAD cells was detected via immunoprecipitation (IP). **D** Co-IP was conducted in LUAD cells transfected with Flag-CHAC1 lentivirus. **E** IP was conducted in LUAD cells transfected with HA-PKM2 lentivirus. **F** CHAC1 and PKM2 co-localization were determined via confocal microscopy scanning of IF staining in LUAD cells. DAPI was used for nuclear staining. Scale bar = 10 µm. **G** Protein levels of PKM2 in overexpression or knockdown of CHAC1 LUAD cells. **H** Protein levels of CHAC1 in overexpression or knockdown of PKM2 LUAD cells. Values below western blot bands are optical density of signal.

Since we have previously provided evidence that CHAC1 increases the glycolytic capacity of LUAD cells, we next investigated the specific mechanism. Silver staining and shotgun MS were performed to detect CHAC1-interacting molecules (Fig. 4A). The isolation of CHAC1-interacting protein complexes from CHAC1 OE A549 cells took place using anti-FLAG monoclonal antibodies and paramagnetic beads. Affinity purification and MS analysis detected 400 binding proteins with CHAC1, with GSEA being mainly enriched in pathways including metabolic processes, glycolytic processes, and splicing-related proteins (Supplementary Tables S6 and S7). We intersected 31 differential expressed proteins in the “glucose metabolism” pathway from RNA-seq with 14 CHAC1-binding proteins in the MS “glycolytic process” and obtained 10 CHAC1- and glycolysis-related proteins (Fig. 4B).

GAPDH and PKM exhibited the highest MS coverage (Supplementary Table S6), and the spectrum of PKM is shown in Supplementary Fig. 4. Since PKM2 is an essential rate-limiting metabolic enzyme in glycolysis [28], we focused on PKM2 for further experiments. The results showed that endogenous PKM2 bound to CHAC1 (Fig. 4C). We transfected an exogenous FLAG-tagged CHAC1 lentivirus and found that CHAC1-FLAG could bind to PKM2 (Fig. 4D). Next, we expressed HA-tagged PKM2 in PC9 and A549 cells and found that FLAG-tagged CHAC1 bound to HA-tagged PKM2 (Fig. 4E). IF analysis revealed that CHAC1 interacted with PKM2 in both the cytoplasm and the nucleus (Fig. 4F).

Given that CHAC1 expression affects PK activity (Fig. 3E), the question is whether this is achieved by affecting PKM2 protein expression. Therefore, we examined PKM2 protein expression by interfering with CHAC1 expression. The results showed that overexpressing or knocking down CHAC1 did not affect PKM2 protein expression (Fig. 4G). Similarly, interfering with PKM2 expression did not affect CHAC1 protein expression (Fig. 4H). These results showed an interaction between CHAC1 and PKM2, which did not change the expression of PKM2 but affected PK activity.

### CHAC1 induces SUMOylation of PKM2 through UBA2/SUMO1 axis

**Fig. 5:**
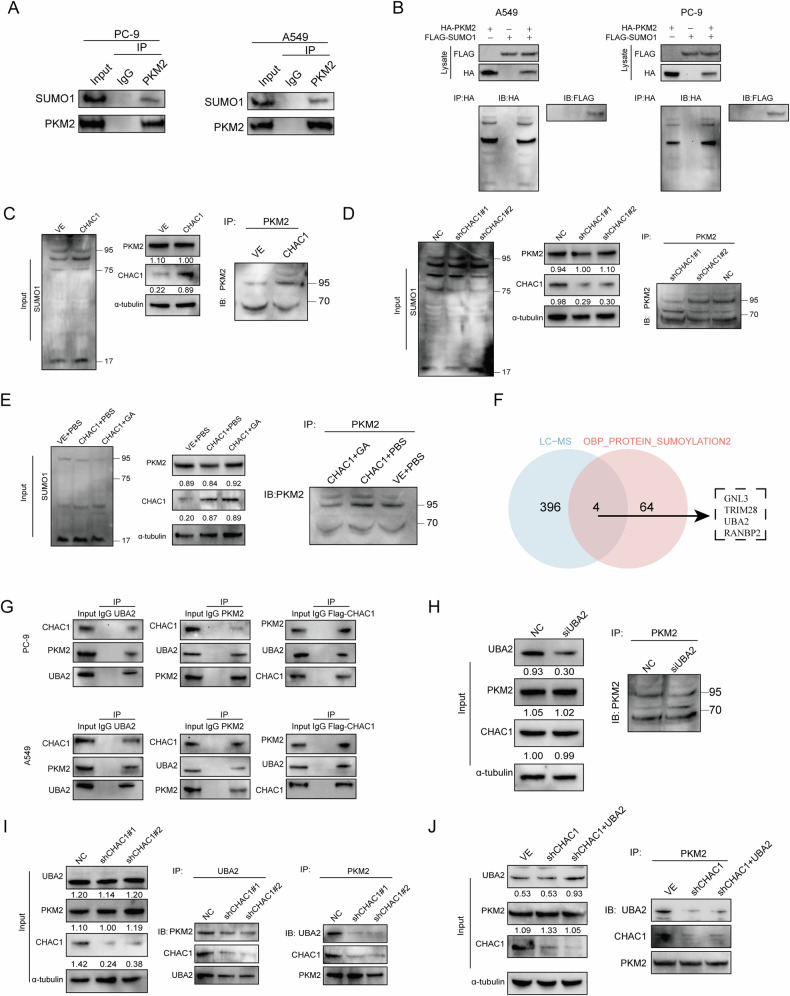
CHAC1 induces SUMOylation of PKM2 protein through UBA2/SUMO1 axis. **A** Endogenous interaction between PKM2 and SUMO1 in LUAD cells was detected via IP. **B** Exogenous interaction between HA-PKM2 and FLAG-SUMO1 in LUAD cells was detected via IP. **C** SUMOylation of PKM2 in CHAC1 overexpression cells was detected via IP. **D** SUMOylation of PKM2 in shCHAC1 cells was detected via IP. **E** SUMO inhibitor GA (50 µm) was added to detect the effect of SUMOylation on PKM2 protein. **F** Intersection of GSEA “GOBP_PROTEIN_SUMOYLATION” signature and LC–MS was used to identify CHAC1-binding proteins affecting PKM2 SUMOylation. **G** IP was used to detect interactions between CHAC1, UBA2, and PKM2. **H** The SUMOylation of PKM2 was detected by knocking down UBA2. **I** The interaction between CHAC1, PKM2, and UBA2 was examined by knocking down CHAC1. **J** The interaction between CHAC1, UBA2, and PKM2 was detected by knockdown of CHAC1 combined with overexpression of UBA2. IB immunoblot, IP immunoprecipitation.

It has been suggested that PKM2 may affect its activity by interfering with its subcellular localization by SUMOylation [29] and SUMO1 promotes the glycolytic ability of tumor cells [30]. Therefore, we explored whether CHAC1 mediates the SUMOylation of PKM2 and further regulates glycolytic metabolism. First, SUMO1 protein was extracted from A549 and PC9 cells for immunoprecipitation and analyzed by liquid chromatography–tandem mass spectrometry (LC–MS/MS). LC–MS/MS analysis revealed that PKM was one of the SUMOylated proteins (Supplementary Fig. 5A). Importantly, endogenous PKM2 SUMOylation in LUAD cells was detected by co-immunoprecipitation (IP). PKM2 SUMOylation was similarly detected when PKM2 (hemagglutinin [HA]) and SUMO1 (Flag) were overexpressed (Fig. 5A, B).

Subsequently, we found that overexpression of CHAC1 resulted in a distinct shift band of PKM2 at 95 kDa. Knockdown of CHAC1 reduced the intensity of the 95 kDa shift band (Fig. 5C, D). SUMOylation of PKM2 resulted in a shift band at 95 kDa [31]. Therefore, to demonstrate that PKM2 undergoes SUMOylation, we added Ginkgolic acid (GA), a small molecule that inhibits SUMOylation by targeting the SUMO-activating enzyme E1 [32], while overexpressing CHAC1. As shown in Fig. 5E, GA inhibited the distinct shift band of PKM2 at 95 kDa caused by CHAC1 overexpression, further supporting enhanced PKM2 SUMOylation. These results indicate that CHAC1 promotes the binding of PKM2 to the SUMO1 protein and its SUMOylation.

Next, we explored whether SUMOylation-related proteins were presented in the LC–MS analysis. The intersection of LC–MS and the “GOBP_PROTEIN_SUMOYLATION” signature in GSEA was taken, and four potential CHAC1-binding proteins that affected PKM2 SUMOylation were identified (Fig. 5F). UBA2 is a component of SUMO-activating enzyme 1 (SAE1), which regulates the critical activation of SUMO proteins. IP experiments revealed an interaction between FLAG-CHAC1, UBA2, and PKM2 (Fig. 5G). Therefore, we speculated that CHAC1 interacts with UBA2 to promote the SUMOylation of PKM2. IF staining showed that CHAC1 co-localized with UBA2 in the cytoplasm (Supplementary Fig. 5B). When we knocked down UBA2, the SUMOlytion level of PKM2 decreased (Fig. 5H and Supplementary Fig. 5C). Then we further examined their interactions, the interaction between UBA2 and PKM2 was significantly attenuated by CHAC1 knockdown (Fig. 5I). The interaction between UBA2 and PKM2 was not restored by UBA2 overexpression CHAC1-knockdown cells (Fig. 5J). These results suggest that CHAC1 may act as a bridge connecting UBA2 and PKM2, thereby facilitating their interaction and enhancing PKM2 SUMOylation.

### CHAC1 regulates translocation of PKM2 into nucleus via SUMOylation

**Fig. 6:**
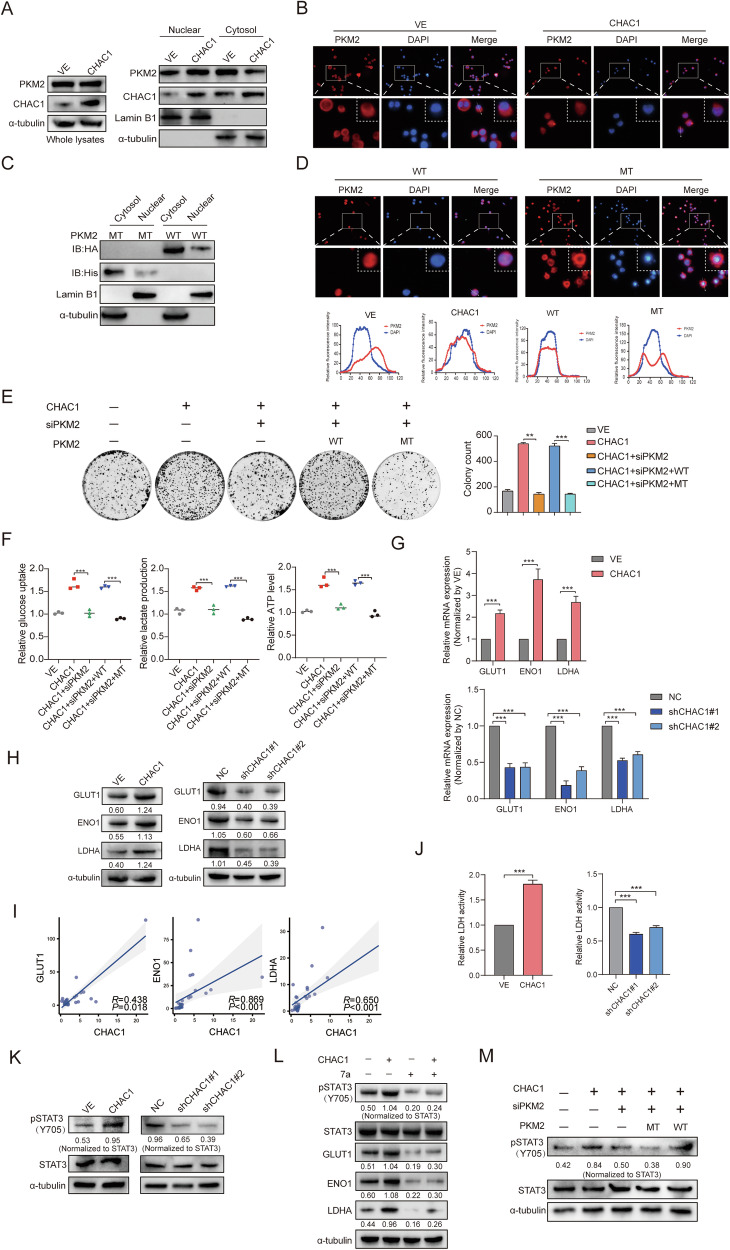
CHAC1 regulates the nuclear translocation of PKM2 via SUMOylation. **A** Nuclear and cytosolic protein lysates prepared from A549 cells stably expressing mock or CHAC1 were assayed via western blotting. **B** Subcellular localization of PKM2 in LUAD cells that stably expressed mock or CHAC1 OE was observed via IF. **C** Cells were transfected with HA-tagged PKM2 (WT), His-tagged PKM2^I267&268A^ (MT), and PKM2 localization was detected by using western blot. **D** Subcellular localization of PKM2 in LUAD cells transfected with PKM2 WT and PKM2 MT was observed via IF. **E**, **F**, **M** PKM2 WT or PKM2 MT were transfected into LUAD cells simultaneously overexpressing CHAC1 and siPKM2, respectively. **E** Colony-formation assay was used to detect cell proliferation. **F** Relative glucose uptake, lactate production, ATP level were detected. **G**, **H** RT-qPCR and western blot were used to detect relative mRNA and protein levels of GLUT1, ENO1, and LDHA in CHAC1 OE and knockdown cells. **I** The correlation between CHAC1 expression and the expression of GLUT1, ENO1, and LDHA in 30 LUAD tissue samples. **J** Detect LDH activity in LUAD cells using a lactate dehydrogenase assay kit. **K** Western blot was used to detect phosphorylation of STAT3^Y705^ and histone H3^T11^. **L** The phosphorylation level of STAT3 was detected after the addition of 7a. **M** Western blot was used to detect phosphorylation of STAT3^Y705^ and histone H3^T11.^ Results are shown as means ± SD. Values below western blot bands are optical density of signal. **p < 0.01, ***p < 0.001.

SUMO-interacting motif (SIM) is a SUMOylation recognition motif of target proteins. There are three potential SIM sites in PKM2, among which IKII265-268 is mutated to PKM2^I267&268A^ (MT), eliminating the SUMO1-induced SUMOylation of PKM [33], which affects PKM2 dimer formation and nuclear translocation [29]. Hence, we hypothesized that CHAC1 regulates PKM2 SUMOylation and nuclear translocation and that PKM2 acts as both a protein kinase and a co-transcription factor in the nucleus. We examined the localization of PKM2 in CHAC1-overexpression cells. Nucleocytoplasmic separation experiments showed that CHAC1 overexpression enhanced the expression level of PKM2 in the nucleus but did not change the overall PKM2 protein level (Fig. 6A). Similar results were obtained with the IF assay (Fig. 6B). When transfected with the PKM2 WT plasmid, PKM2 nuclear translocation was enhanced, whereas transfection with the PKM2 MT plasmid abolished this effect (Fig. 6C, D). These results indicate that CHAC1 modifies specific sites through SUMOylation to promote the nuclear translocation of PKM2.

To elucidate whether the progression induced by CHAC1 depends on the nuclear translocation of PKM2. We stably transfected a CHAC1-overexpressing lentivirus in cells with PKM2 knockdown. Results showed that PKM2 knockdown significantly reduced cell proliferation and migration, and CHAC1 overexpression could not reverse these effects (Supplementary Fig. S6A, B). These results suggest that PKM2 is a key downstream effector of CHAC1 during the regulation of cell proliferation and metastasis.

Then, we transfected siPKM2, PKM2 WT, and PKM2 MT plasmids into cells stably overexpressing CHAC1. The results showed that PKM2 inhibited the enhanced cell proliferation, migration, and invasion abilities enhanced by CHAC1. More importantly, these malignant phenotypes could be rescued by reintroducing PKM2 WT but not PKM2 MT (Fig. 6E and Supplementary Fig. S6C, D). This indicates that the effects of CHAC1 on cell proliferation and metastatic capacity depend on the nuclear translocation of PKM2.

Nuclear PKM2 acts as both a protein kinase and a co-transcription factor, promoting the glycolytic process in tumors [20]. We initially assessed the correlation between CHAC1 expression and downstream glycolytic genes regulated by PKM2. Bioinformatic analysis revealed a positive correlation between CHAC1 expression and GLUT1 (*R* = 0.174, *p* < 0.001) and LDHA (*R* = 0.088, *p* = 0.040, Supplementary Fig. S6E). ENO1 was also correlated with CHAC1 but not significantly (Supplementary Fig. S6E). Upon examining the expression of glycolytic genes in cells, we observed that CHAC1 overexpression significantly upregulated the mRNA and protein levels of GLUT1, ENO1, and LDHA. Conversely, the knockdown of CHAC1 led to a downregulation of these glycolytic genes (Fig. 6G, H). Further analysis using human LUAD tissue samples demonstrated a significant positive correlation between CHAC1 and GLUT1 (*R* = 0.438, *p* = 0.018), ENO1 (*R* = 0.869, *p* < 0.001), and LDHA expression (*R* = 0.650, *p* < 0.001, Fig. 6I). Notably, as LDH catalyzing the conversion of pyruvate to lactate, CHAC1 overexpression also resulted in increased LDH activity (Fig. 6J).

Given that PKM2 acts as a co-transcription factor, potentially interacting with transcription factors (TFs) like c-Myc in the nucleus to influence target gene regulation [20], we investigated whether c-Myc expression was affected. Our results indicate that CHAC1 overexpression promotes c-Myc mRNA expression; however, it does not significantly alter c-Myc protein levels or its distribution between the nucleus and cytoplasm (Supplementary Fig. S6F, G). This suggests that nuclear PKM2 may regulate glycolytic gene expression through other mechanisms.

STAT3 phosphorylation has been reported as a known substrate of PKM2 when functioning as a protein kinase [34, 35]. Activated STAT3, as a TF, regulates the expression of glycolytic genes such as GLUT1, ENO1, and LDHA. Thus, we examined the effect of CHAC1 on the phosphorylation of STAT3. Our findings indicated that overexpression of CHAC1 increased the phosphorylation of STAT3^Y705^, whereas knockdown of CHAC1 significantly reduced the levels of phosphorylated STAT3^Y705^ (Fig. 6K). The selective STAT3 inhibitor 7a, which blocks STAT3^Y705^ phosphorylation [36], was co-administered with overexpressed CHAC1, resulting in a marked suppression of GLUT1, ENO1, and LDHA protein expression (Fig. 6L). This confirms that CHAC1’s regulation of glycolytic genes is mediated via STAT3^Y705^ phosphorylation.

We explored the effect of the PKM2 MT on STAT3^Y705^ phosphorylation. The results showed that PKM2 downregulation blocked the CHAC1-induced increase in the phosphorylation of STAT3^Y705^ (Fig. 6M). Similar results were observed for the transcription of PKM2 target genes (Supplementary Fig. 6H). Collectively, these results suggest that nuclear PKM2-mediated glycolysis is critical for CHAC1 to promote LUAD progression and metastasis.

### E2F Transcription Factor 1 (E2F1) activates CHAC1 transcription in LUAD cells

**Fig. 7:**
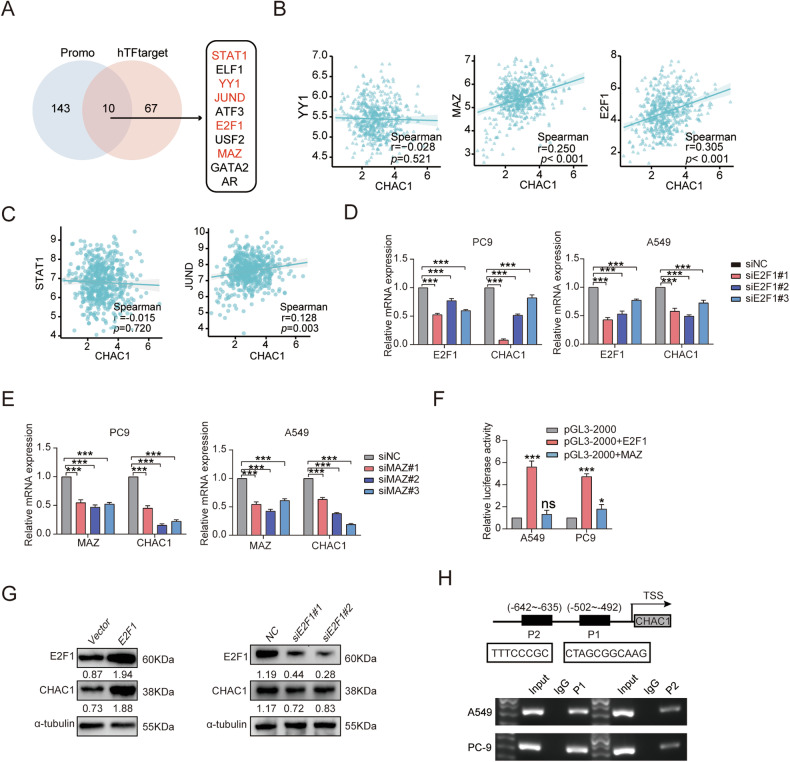
E2F Transcription Factor 1 (E2F1) activates CHAC1 transcription in LUAD cells. **A** Screening transcription factors (TFs) that may act on CHAC1 promoter by PROMO and hTFtarget. **B**, **C** Analyze the correlation between TFs and CHAC1 expression. **D**, **E** E2F1 siRNA or MAZ siRNA were transfected to detect CHAC1 mRNA expression. **F** Dual-luciferase reporter assay was used to analyze the effect of E2F1 or MAZ plasmid transfection on the fluorescence activity of CHAC1 promoter. **G** Detect the protein expression of CHAC1 in E2F1 overexpressing or knockdown cells. **H** ChIP-qPCR assay detects the fragments that E2F1 may bind to CHAC1. Results are shown as means ± SD. Values below western blot bands are optical density of signal. *p < 0.05, ***p < 0.001, ns no statistical significance.

To elucidate the mechanism of CHAC1 overexpression in LUAD, we performed promoter analysis to investigate whether CHAC1 expression is regulated by certain TFs. The PROMO [37, 38] and hTFtarget [39] databases were used to mine potential regulators, and ten TFs with high binding potential were obtained (Fig. 7A). Then, JASPAR database was utilized to analyze the potential binding of TFs to CHAC1 [40]. For the JASPAR score, higher scores were observed for YY1, MAZ, E2F1, STAT1, YY1, and JUND (score > 8) (Supplementary Table S8). Correlation analysis showed that MAZ (*r* = 0.250, *p* < 0.001), E2F1 (*r* = 0.305, *p* < 0.001), and CHAC1 were moderately positively correlated (Fig. 7B, C). After MAZ and E2F1 knockdown, CHAC1 mRNA expression was downregulated (Fig. 7D, E).

To explore whether there is a direct binding interaction between E2F1, MAZ, and the CHAC1 promoter, we inserted a CHAC1-containing promoter fragment into a reporter plasmid. The plasmid containing the CHAC1 promoter was co-transfected with E2F1- and MAZ-overexpression plasmids, and E2F1 significantly increased the relative luciferase activity in LUAD cells (Fig. 7F). Moreover, the overexpression of E2F1 promoted CHAC1 protein expression, whereas knocking down E2F1 had the opposite effect (Fig. 7G). JASPAR software predicted two possible binding sites for E2F1 on CHAC1 (Fig. 7H), and the ChIP-qPCR assay showed that the P1 fragment of E2F1 had a strong possibility of binding to the CHAC1 promoter (Fig. 7H). These results suggested that E2F1 activates CHAC1 transcription in LUAD.

### CHAC1 promotes progression of LUAD in vivo

**Fig. 8:**
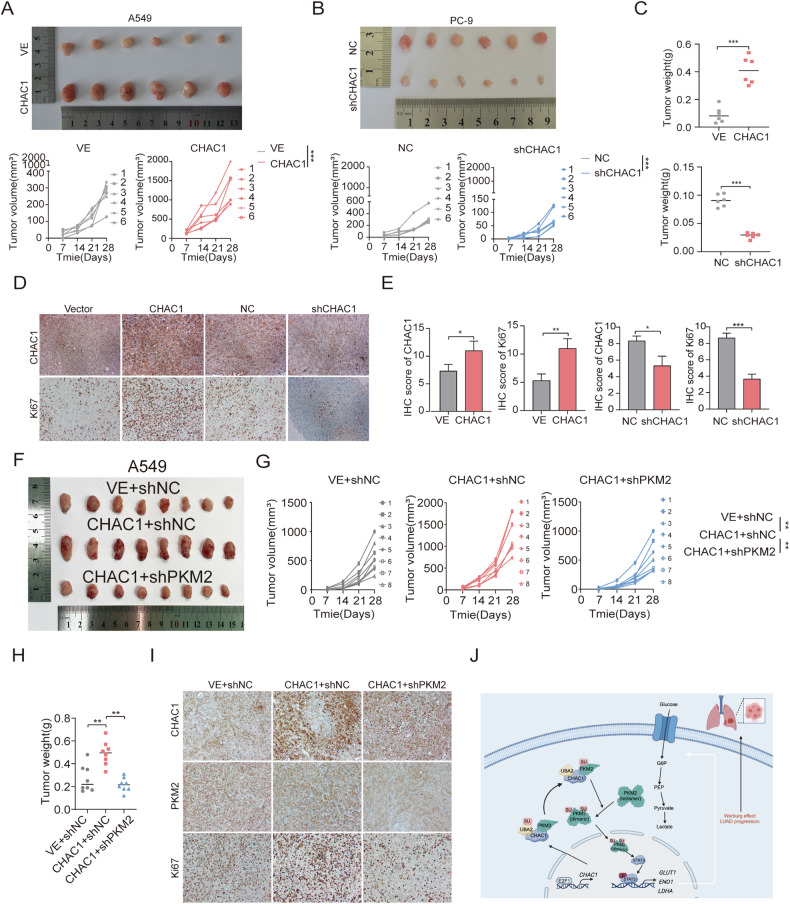
CHAC1 promotes LUAD growth in vivo. **A**–**C** In vivo growth of indicated cell lines stably overexpression or silencing CHAC1 were examined (*n* = 6). Xenograft volume (cm) and weight (g) were measured. Tumor volume was calculated by 1/2 (length × width^2^). Data are expressed as means ± SD. Significant difference was based on unpaired *t*-test. **D**, **E** Immunohistochemistry staining and IHC scores of CHAC1 and Ki67 in respective xenograft tumor tissues. **F**–**H** In vivo growth of A549 cells stably co-transfected with CHAC1 OE and shPKM2 lentivirus (*n* = 6). Xenograft volume (cm) and weight (g) were measured. **I** Immunohistochemistry staining of CHAC1, PKM2, and Ki67 in respective xenograft tumor tissues. **J** Working model illustrating that CHAC1 promotes glycolysis and progression of LUAD by regulating PKM2 SUMOylation. *p < 0.05, **p < 0.01, ***p < 0.001, ns no statistical significance.

To explore the association between CHAC1 and LUAD development in vivo, A549 and PC9 cells were transfected with CHAC1 overexpression and shCHAC1 lentiviruses, respectively. Subsequently, these cells were injected into nude mice to construct a xenograft neoplasm system. As revealed by the findings, CHAC1 overexpression significantly increased the volume and weight of tumors in the mice (Fig. 8A, B) and vice versa (Fig. 8C). In addition, after the CHAC1 was overexpressed, the expression level of the proliferation marker Ki67 was upregulated, whereas the knockdown of CHAC1 had the opposite effect (Fig. 8D, E).

We further analyzed whether CHAC1/PKM2 axis in vivo affected orthotopic xenograft tumor growth. First, a PKM2-knockdown lentivirus was constructed in A549 cells. Among them, the knockdown efficiency of shPKM2#1 was the highest, and this lentivirus was used for subsequent experiments (Supplementary Fig. S7A). In the subcutaneous xenograft model, CHAC1- and shPKM2-expressing cells inhibited LUAD orthotopic xenograft tumor growth when compared with CHAC1-expressing cells (Fig. 8F–H). Moreover, IHC showed that the expression of Ki67 in the CHAC1- and shPKM2-expressing groups was lower than that in the CHAC1-expressing group (Fig. 8I and Supplementary Fig. S7B). Taken together, our findings suggest that CHAC1/PKM2 axis is essential for LUAD progression.

## Discussion

The biological role and molecular mechanism of action of CHAC1 in LUAD remain unclear. In the current study, we demonstrated that the TF E2F1 activates CHAC1 transcription, resulting in the upregulation of CHAC1 mRNA and protein expression. CHAC1 functions as a bridge connecting UBA2 and PKM2, leading to the induction of PKM2 SUMOylation. Furthermore, SUMO-modified PKM2 nuclear translocation activates the transcription of proliferation- and glycolysis-related genes, and CHAC1 promotes LUAD glycolysis and tumor progression.

CHAC1 plays an important role in determining cell fate. First, CHAC1 prevents the glutathione peroxidase 4–reduced GSH system, leading to the lethal accumulation of lipid peroxides on the cell membrane and ferroptosis [41]. This has been reported in tumor and non-tumor diseases. CHAC1 acts as a regulatory gene in the “unfolded protein response” pathway and regulates apoptosis [42]. Although these studies suggest that CHAC1 expression correlates with cell fate, the effect of CHAC1 expression on cell fate depends on the cell type. High CHAC1 expression is associated with poor prognosis in breast cancer [5, 6], ovarian cancer [43], renal hyaline carcinoma [7], and uveal melanoma [8]. The opposite is true for the liver [11], gastric [10], prostate [9], and oral squamous cell carcinomas [44]. However, current studies have mainly focused on bioinformatic analysis and not in-depth mechanistic studies, and the role of CHAC1 in lung cancer progression remains unclear. In this study, we demonstrated for the first time that CHAC1 expression is upregulated in LUAD and is correlated with its malignant progression.

To investigate the mechanism of action of CHAC1 in LUAD, we defined the upstream promoter sequence of CHAC1 and confirmed that E2F1 is a TF that upregulates CHAC1 in LUAD. E2F1 is a member of the E2F TF family, which can activate or silence the transcription of various genes and is an important regulatory factor in cell proliferation, differentiation, apoptosis, and drug resistance. As the first member of the E2F family, E2F1 acts as a transcriptional cofactor and interacts with retinoblastoma proteins to regulate the transcription of cell-cycle-related proteins [45]. E2F1 also serves as a TF that plays an important role in cancer progression. In HCC, E2F1 upregulates the RNA-binding protein AUF1, promoting HCC development and chemoresistance [46]. In ccRCC, overexpression of E2F1 upregulates SREBP1-induced abnormal lipid metabolism, increasing ccRCC proliferation and metastasis [47]. Here, we verified that E2F1 directly binds to the CHAC1 promoter region and activates its transcription, thereby upregulating CHAC1 mRNA and protein expression.

Currently, the underlying molecular mechanism of action of CHAC1 in LUAD progression is unclear. We used bioinformatic analysis, RNA-seq, and LC–MS to detect signaling pathways and interacting proteins that the abnormal expression of CHAC1 may influence. In the present study, we report that CHAC1 affects PK activity but not PKM2 protein expression. PK is the rate-limiting enzyme in glycolysis and catalyzes the conversion of PEP to PE. PKM encodes two PK isoforms, PKM1 and PKM2, via alternative splicing [48]. Among these, PKM1 exists only as a highly active tetramer, whereas PKM2 switches between that and a low-activity dimer [49]. The low catalytic activity of the dimer PKM2 increases the production of glycolytic intermediates [49, 50]. Changes in the PK enzymatic activity are associated with Y105 phosphorylation [34], alternative splicing of transcripts [51], posttranslational modifications [52, 53], and hypoxia [54]. We demonstrated that CHAC1 can induce the SUMOylation of PKM2 and promote the nuclear translocation of PKM2 dimers. Re-localization of PKM2 to the nucleus suggests it plays a crucial role in this process. In our study, nuclear PKM2 acted as a protein kinase, promoting the phosphorylation of STAT3, thereby regulating the expression of downstream glycolysis-related genes such as GLUT1, ENO1, and LDHA, which in turn promotes tumor progression. This is consistent with the findings of Zhao et al. [33, 34].

The SAE1/UBA2 heterodimer is involved in SUMOylation and is a key kinase that controls SUMO protein activity. SUMOylation is involved in glycolytic metabolism, and UBA2 knockdown inhibits SUMO protein activation and maintains high PK activity, thereby reducing glycolytic metabolism in rheumatoid arthritis [27]. In HCC, UBA2 activates SUMO1, promotes PKM2 nuclear translocation, and activates EMT and the STAT3 signaling pathway to promote HCC progression [55]. In this study, we revealed that CHAC1 interacted with UBA2 but did not affect UBA2 expression, and that CHAC1 maintained low PK activity and dimer PKM2 nuclear transfer. The phosphorylation of PKM2 at Y105 induces the formation of inactive tetrameric PKM2 dimers and decreases PK activity [53]. SAE1/UBA2-mediated SUMOylation blocks PKM2 phosphorylation and increases PK activity [27]. Therefore, this was not validated in this study.

In this study, the overexpression of CHAC1 caused downregulation of PK activity and unchanged PKM2 protein expression. PKM2 may function mainly as a protein kinase to regulate gene transcription in the nucleus. PKM2 SIM IKII265-268 site mutation eliminates SUMO1-induced PKM2 SUMOylation, which affects PKM nuclear translocation [31, 33]. In the present study, transfection with the PKM2 WT plasmid increased PKM2 nuclear translocation, whereas transfection with the PKM2 MT plasmid failed to induce PKM2 nuclear translocation. Importantly, the PKM2 WT plasmid enhanced the PKM2-dependent Warburg effect. Our study provides evidence for a model in which CHAC1 promotes PKM2 nonmetabolic enzyme activity by promoting PKM2 SUMOylation and nuclear translocation.

This study conducted an in-depth mechanistic investigation of the role of CHAC1 in LUAD; however, there are still limitations to this study. Transgenic models with CHAC1 knockouts may better determine the role of CHAC1 in LUAD. This model is expected to be used for further verification in the future.

Taken together, we confirm that CHAC1 is a key target of the Warburg effect, promoting the malignant progression of LUAD. E2F1 directly targets the CHAC1 promoter, efficiently activating CHAC1 transcription in LUAD. CHAC1 acts as a bridge connecting UBA2 and PKM2, facilitating PKM2 SUMOylation and its translocation from the cytoplasm to the nucleus. Additionally, PKM2 functions as a protein kinase, activating the STAT3 signaling pathway and upregulating the transcription of genes associated with glycolysis, ultimately leading to cell proliferation and metastasis (Fig. 8J). Our study indicates that targeting CHAC1 or blocking the CHAC1/UBA2/PKM2 axis may be a promising treatment strategy for LUAD.

## Supplementary information

Supplementary Data

Supplementary Data

Supplementary Data

Supplementary Data

## Data Availability

All data associated with this study are presented in the article or the Supplementary Materials and Methods.

## Materials availability

The materials that support the findings of this study are available from the corresponding authors upon reasonable request.
