# Correction: CHAC1 blockade suppresses progression of lung adenocarcinoma by interfering with glucose metabolism via hijacking PKM2 nuclear translocation

**DOI:** 10.1038/s41419-024-07235-y

**Published:** 2024-12-09

**Authors:** Junfan Pan, Sixuan Wu, Qihong Pan, Yuan Zhang, Liu He, Qiwei Yao, Jinyuan Chen, Jiancheng Li, Yiquan Xu

**Affiliations:** 1 Clinical Oncology School of Fujian Medical University, Fujian Cancer Hospital, Fuzhou, China; 2 Department of Radiation Oncology, Fujian Cancer Hospital, Fuzhou, China; 3 The First Affiliated Hospital of Xiamen University, Xiamen, China; 4 School of Basic Medical Sciences, Fujian Medical University, Fuzhou, China; 5 The Central Laboratory, Fujian Key Laboratory of Precision Medicine for Cancer, The First Affiliated Hospital, Fujian Medical University, Fuzhou, China; 6 Department of Thoracic Oncology, Fujian Cancer Hospital, Fuzhou, China

**Keywords:** Cancer metabolism, Tumour biomarkers

Correction to: *Cell Death and Disease* 10.1038/s41419-024-07114-6, published online 05 October 2024

Upon reviewing our published work, we discovered an unintentional duplication in Figure 8D, where the Ki67 immunohistochemistry images for the vector group and the shCHAC1 group appear identical. This was an inadvertent copy-paste error that occurred during the image assembly process facilitated by Adobe Illustrator software, for which we take full responsibility. We have promptly revised Figure 8D to reflect the correct data. The incorrect use of the image does not affect our conclusion.
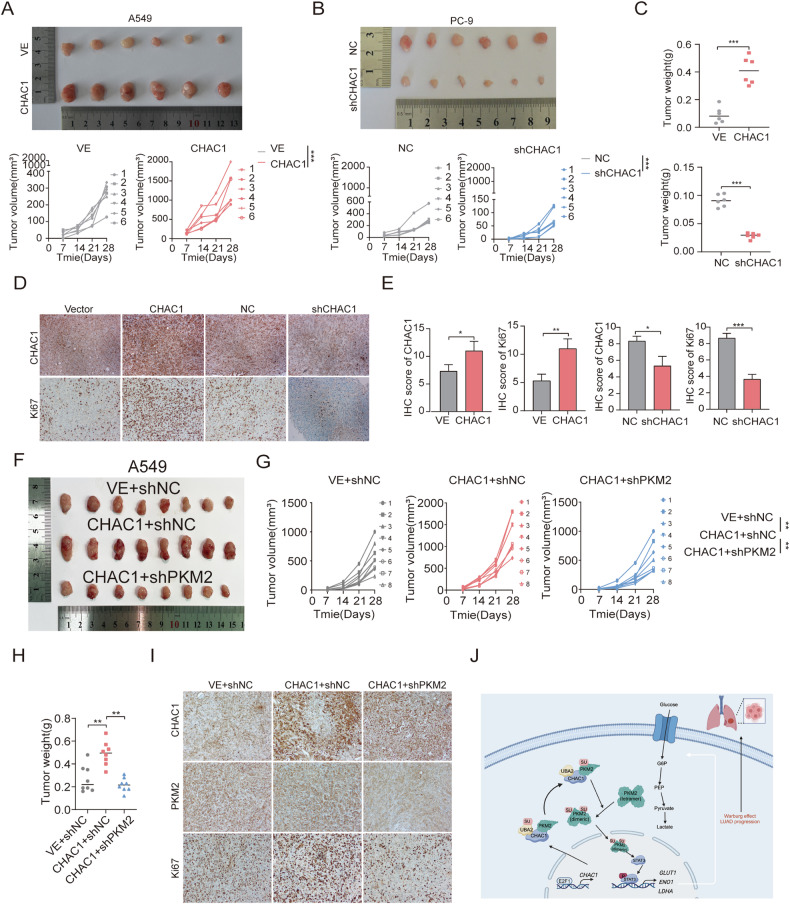

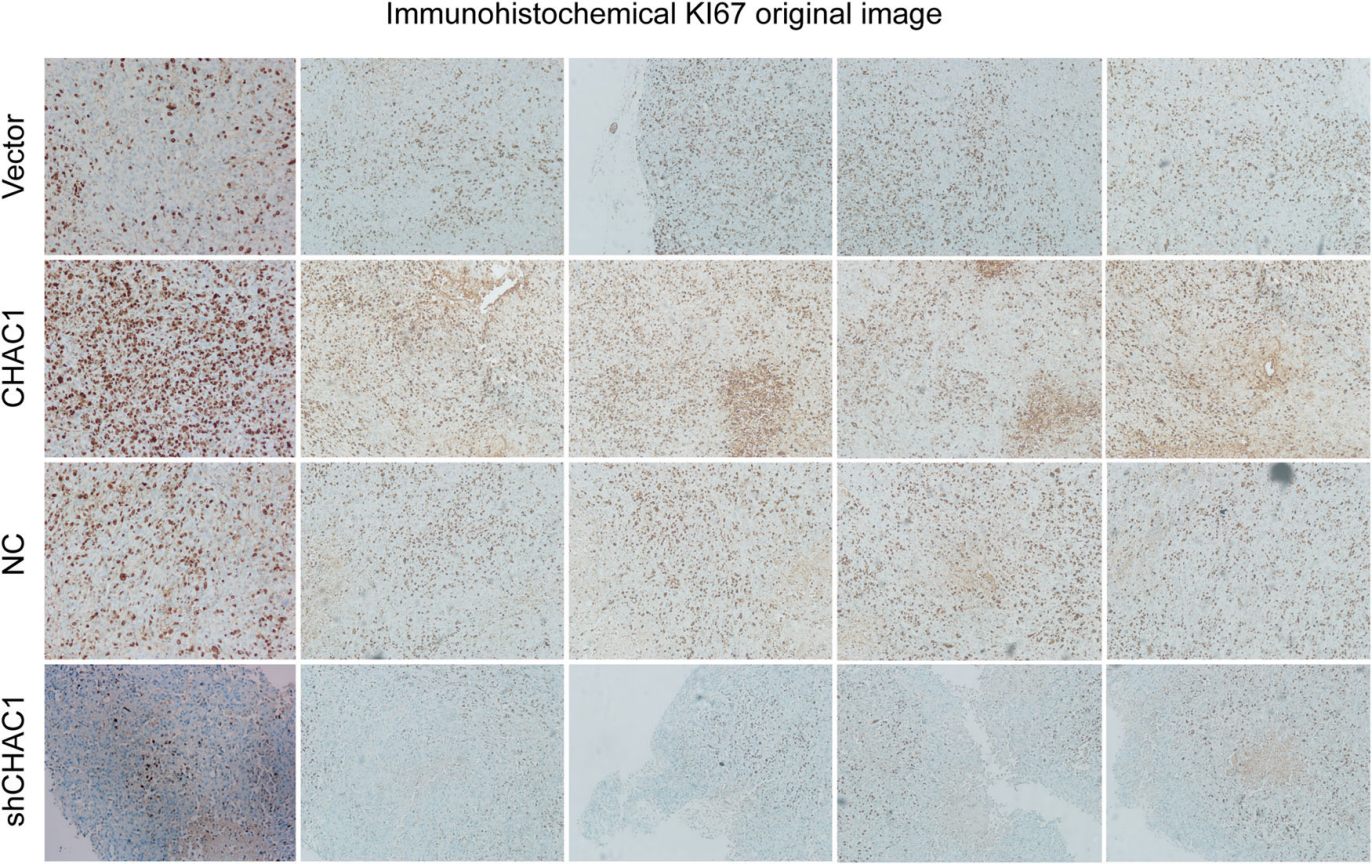


The original article has been corrected.

