# Supplementary material for: CHAC1 blockade suppresses progression of lung adenocarcinoma by interfering with glucose metabolism via hijacking PKM2 nuclear translocation
Source: Cell Death Dis. 2024 Oct 5;15(10):728. doi: 10.1038/s41419-024-07114-6 (PMC11455913; doi:10.1038/s41419-024-07114-6)
Supplement: Supplementary file 1 — Supplementary Data [file 41419_2024_7114_MOESM1_ESM.docx]

**Supplementary Data**

**CHAC1 blockade suppresses progression of lung adenocarcinoma by interfering with glucose metabolism via hijacking PKM2 nuclear translocation**

Junfan Pan^1,2,7^, Sixuan Wu ^1,2,7^, Qihong Pan^1,2,7^, Yuan Zhang^3^ , Liu He^4^ , Qiwei Yao^1,2,^ ^✉^, Jinyuan Chen^5,✉^, Jiancheng Li^1,2,✉^and Yiquan Xu^1,6,✉^

Correspondence to: E-mail: [xuyiquan1018@fjmu.edu.cn](mailto:xuyiquan1018@fjmu.edu.cn); [jianchengli_jack@126.com](mailto:jianchengli_jack@126.com); cjyfjmu@163.com; yqwviva@126.com.

**This PDF file includes:**

Materials and Methods

Supplementary Fig.1 to 7

Supplementary Table S1 to S5, S8

**Other Supplementary Materials for this manuscript include the following:**

Supplementary Table S6 to S7

Materials and Methods

**Cell culture**

The human LUAD cell lines (A549,PC9 H1975,H1299) were purchased from the Cell Room of the School of Medicine of Central South University. Human LUAD cell line Hcc827 and human normal lung epithelial cells Beas-2B were purchased from procell (Wuhan, China). Cells were cultured with RPMI-1640 medium supplemented with 10% fetal bovine serum (FBS) ,100 U/mL penicillin and 100 μg/mL streptomycin in a saturated humidity atmosphere of 5% CO2 at 37°C. All cancer cell lines were authenticated using short tandem repeat profiling and were tested for mycoplasma contamination and confirmed to be negative.

**Quantitative real-time PCR (qPCR)**

Total RNA was extracted from treated LUAD cells using TRIzol (Invitrogen). Then, cDNA was synthesized from total RNA using the PrimeScript RT kit (TAKARA) according to the manufacturer's protocol. Quantitative PCR(q-PCR) was used to detect mRNA expression. The comparative threshold cycle (2-ΔΔCt) method was used to detect gene expression levels. The primer sequences used are listed in Supplementary Table S1**.** Expression levels were normalized to that of the housekeeping gene ACTIN, and each sample was assessed in triplicate.

**Western blotting**

Proteins in cells were treated with RIPA lysates containing 1%protease inhibitor cocktail (P1005, Beyotime, China) and phosphatase inhibitor cocktail (P1081, Beyotime). China) were used for extraction. Cytoplasmic and nuclear proteins were extracted using NE-PER extraction reagent (Thermo, USA ). Protein concentration was determined by bicinchoninic acid assay (Beyotime Biotechnology). Total cell lysates were separated by 4% to 20% sodium dodecyl sulfate-polyacrylamide gel electrophoresis (SDS-PAGE) and transferred to PVDF membranes. The membranes were blocked with TBST buffer containing 5% skim milk powder for 1 h, after which the membranes were incubated with the recommended dilution ratio of primary antibodies overnight at 4 ° C. Subsequently, PVDF membranes were incubated with horseradish peroxidase (HRP) -conjugated appropriate secondary antibodies for 1 h, and protein bands were visualized with a chemiluminescent HRP substrate and imaging system (Chemidoc, Bio-Rad). The primary and secondary antibodies used were listed in Supplementary Table S2.

**Assessment of cell proliferation**

Colony formation assay was used to determine cell proliferation ability. Transfected A549 and PC9 cells (500 cells per well) were seeded in 6-well plates and cultured under normal conditions for 2 weeks. Cell clones（>50 cells）were fixed with 4% paraformaldehyde and stained with crystal violet (Beyotime), and the number of visible clones was counted under a microscope.

**Lipid peroxidation and cell death** **profiling by flow cytometry**

For lipid peroxidation analysis, 3×10^3^ tumor cells were collected and resuspended in 300 µl PBS containing 5 µM BODIPY 581/591 C11. The cells were then incubated in a tissue culture incubator at 37°C for 20 minutes, washed, and resuspended in 200 µl PBS before being immediately analyzed on a flow cytometer (BD FACSVerse).

For cell death assays, 2-4×10^4 cells, including suspended dying cells, were collected and resuspended in 1 µg/ml 7-Aminoactinomycin D (7-AAD) in 200 µl PBS for 10 minutes. The samples were then immediately analyzed on a flow cytometer, and the 7-AAD positive population was quantified as the percentage of dead cells.

**Glutathione quantification**

Glutathione levels, including reduced glutathione (GSH) and oxidized glutathione (GSSG), in tumor cells were quantified using colorimetry following the manufacturer's instructions (Beyotime, S0053). Cell precipitates were collected by centrifugation, frozen in liquid nitrogen, and stored at -80°C. The supernatant was utilized for determining total glutathione levels.For the assay, 10 µl of the supernatant was mixed with 150 µl of the total glutathione assay working solution. After a 5-minute incubation period, 50 µl of NADPH solution was added, and the absorbance at 532 nm was measured. The total GSH content in the samples was calculated using a GSH standard curve.

**Figures S1-S7**

**Figure. S1.**

**
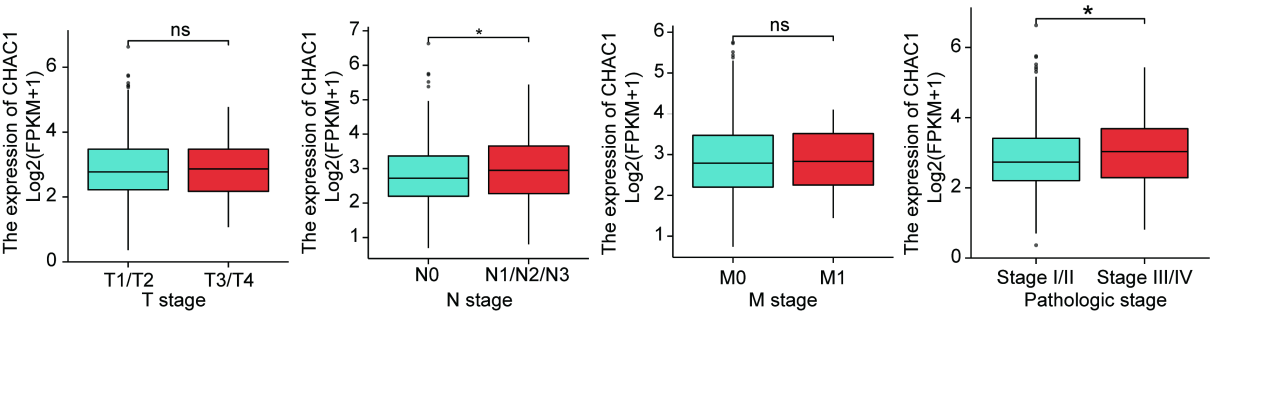
**

**Supplementary Figure 1.** Using TCGA data set to analyze the correlation between CHAC1 expression and Tumor-Node-Metastasis (TNM) stages. ^*^*p* < 0.05, ^**^*p* < 0.01, ^***^*p* < 0.001，ns means no statistical significance.

**Figure. S2.**


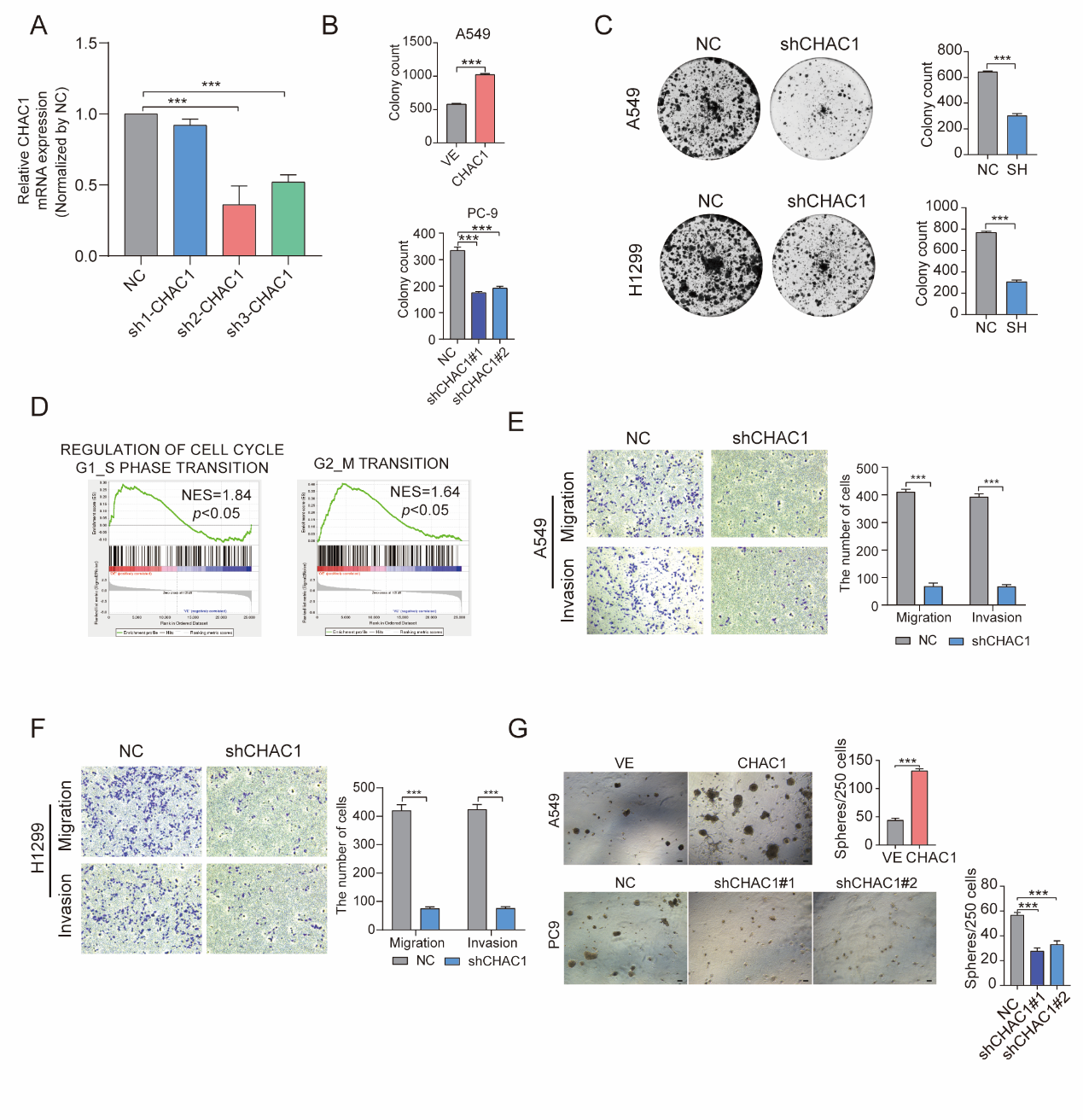
**Supplementary Figure 2.** (A)RT-qPCR were utilized for detecting the knockdown efficiency of CHAC1 in PC-9 cells. (B, C)Colony formation assay, performed to detect effect of overexpression or knockdown of CHAC1 on cell proliferation. (D)Gene set enrichment analysis (GSEA) was used to analyze the enriched pathways of CHAC1 in LUAD. (E, F)Transwell assay were used to detect cell migration and invasion abilities after knockdown of CHAC1. Scale bars, 200 µm. (G)Tumoursphere assay of LUAD cells transfected with overexpression or knockdown of CHAC1. The results are shown as means ±SD.^***^*p* < 0.001.

**Figure. S3.**


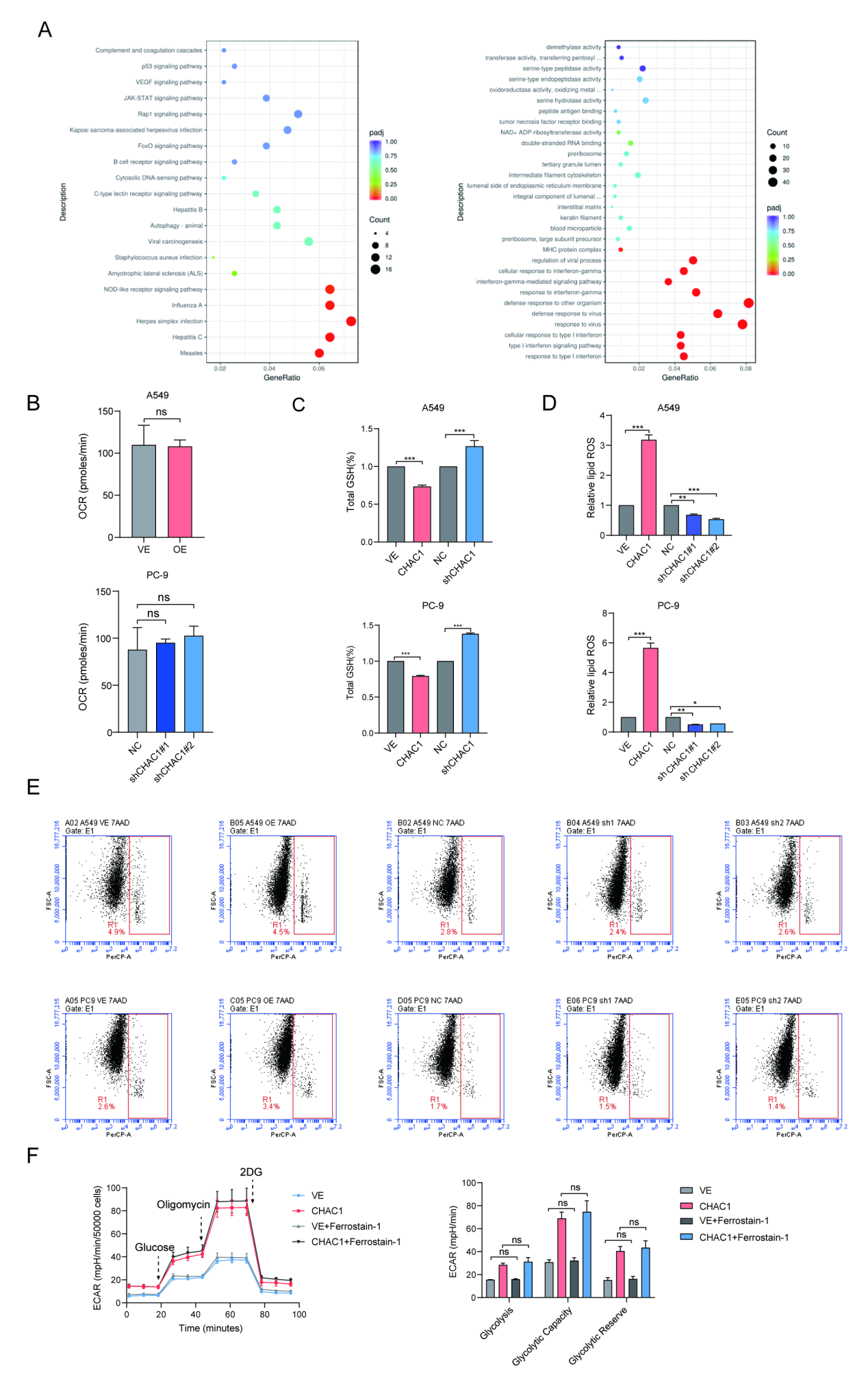


**Supplementary Figure 3.** (A)RNA-seq was performed on CHAC1 overexpression group and control group. KEGG pathway analysis and GO analysis of differentially expressed genes were performed. (B) OCAR of overexpression and knockdown of CHAC1 were monitored. (C, D) Total glutathione content or lipid ROS level were detected in CHAC1 overexpression or knockdown cells. (E) Flow cytometry was used to detect cell death in LUAD cells. (F) Ferrostatin-1 (Fer-1, 10 µM) was added for 20 h to detect the level of ECAR, glycolysis, glycolytic capacity and glycolytic reserve. The results are shown as means ±SD.^***^*p* < 0.001，ns means no statistical significance.

**Figure. S4.**


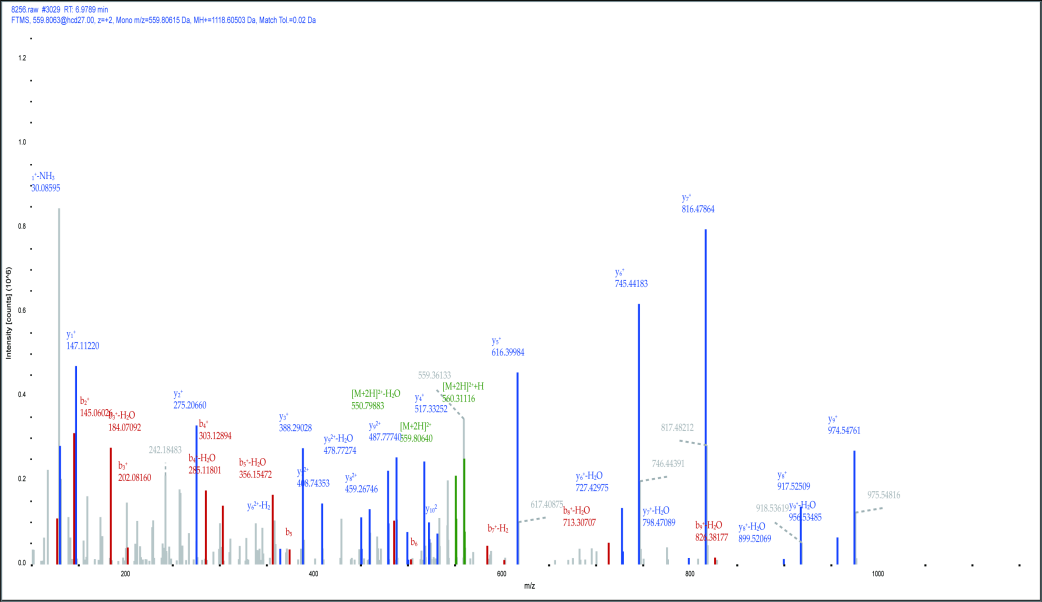


**Supplementary Figure 4.** (A) The liquid chromatography-tandem mass spectrometry (LC-MS/MS) profile of PKM2 was displayed by pull-down CHAC1.

**Figure. S5.**


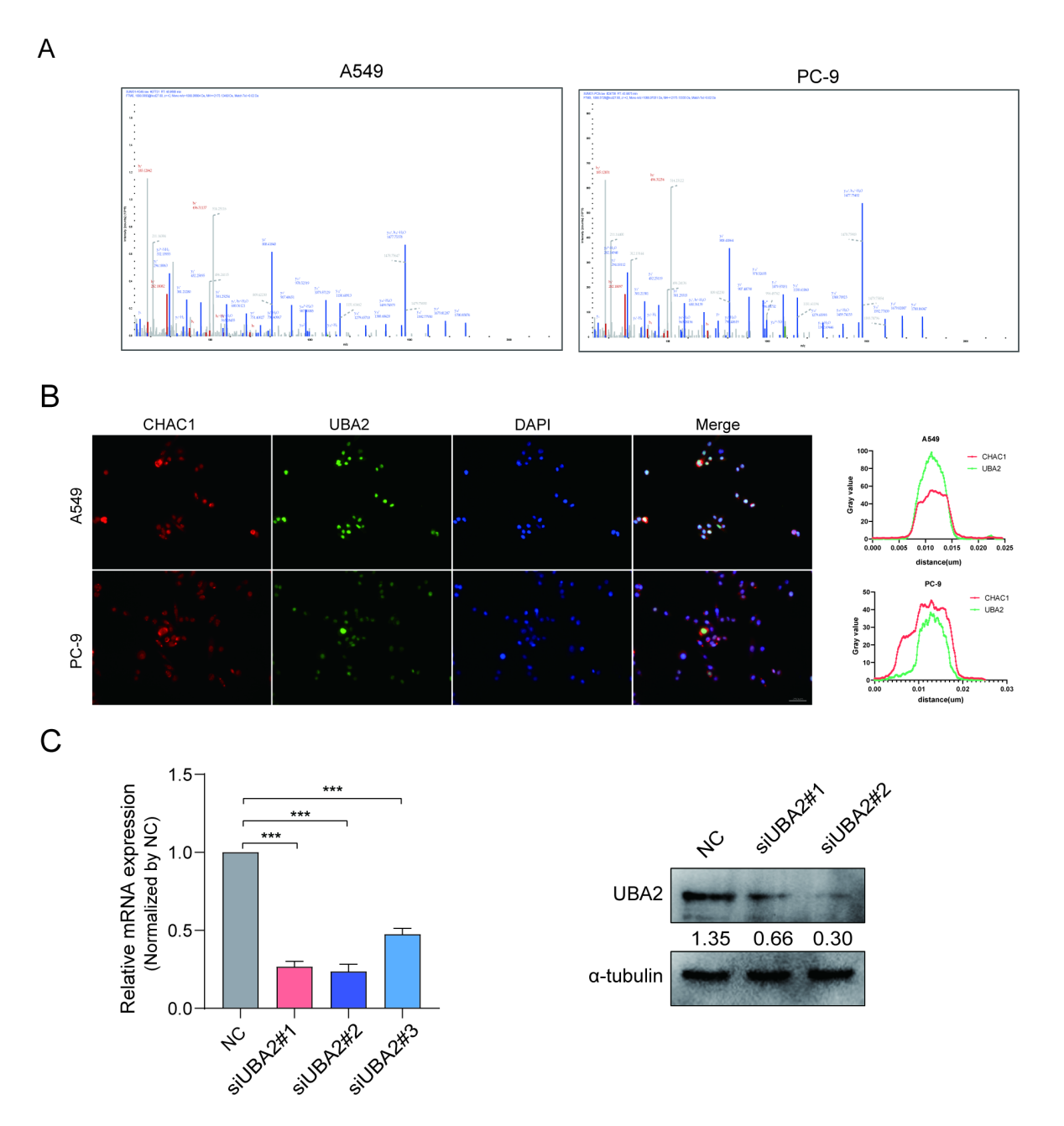


**Supplementary Figure 5.** (A) The liquid chromatography-tandem mass spectrometry (LC-MS/MS) profile of PKM was depicted by pull-down SUMO1. (B) RT-qPCR and Western blot were employed to assess the knockdown efficiency of siUBA2. (C) Immunofluorescence staining was utilized to examine the localization of CHAC1 and UBA2 in cells.The results are shown as means ±SD.^***^*p* < 0.001.

**Figure. S6.**


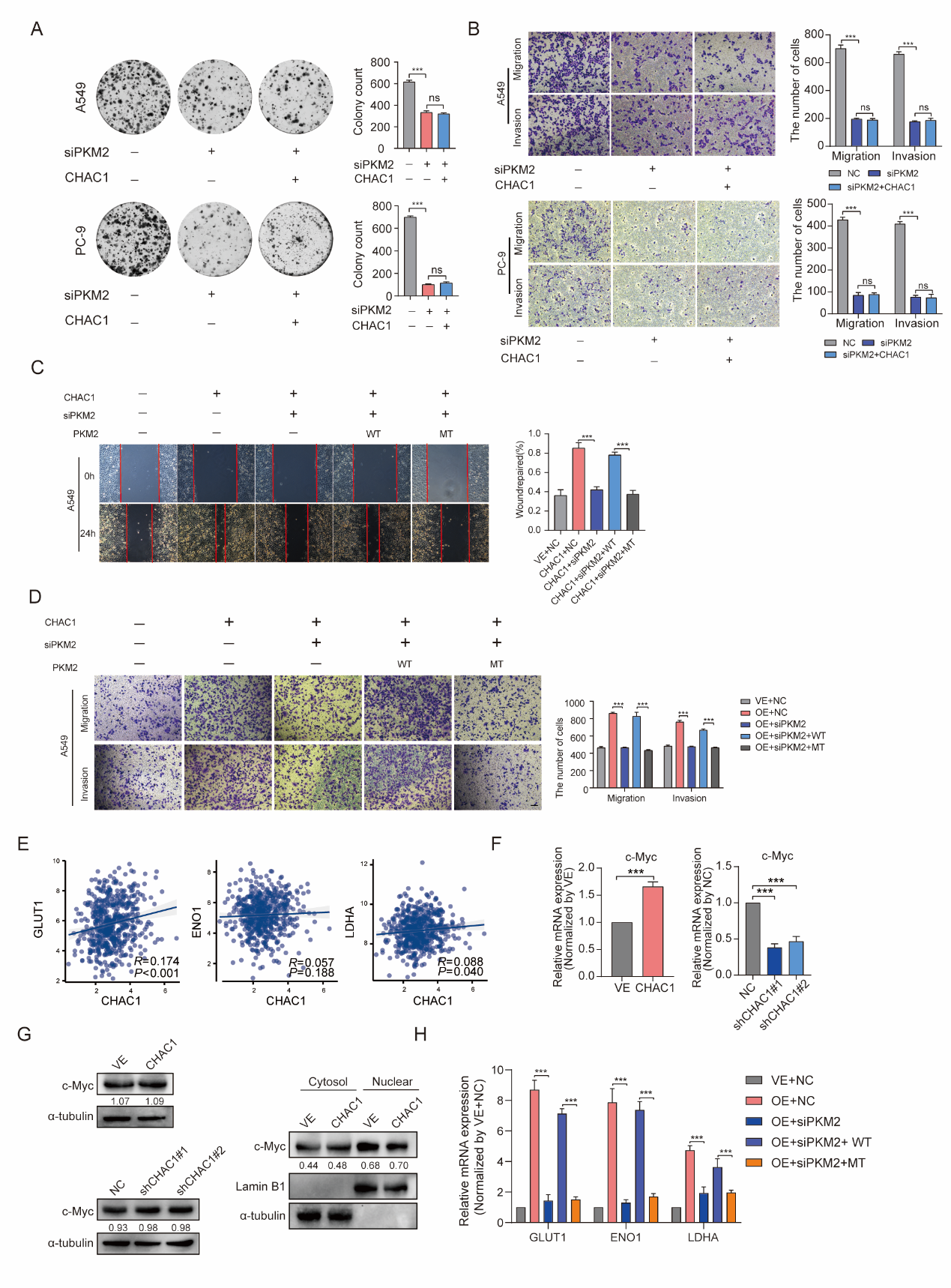


**Supplementary Figure 6.** (A, B) Cell proliferation and migration ability were detected in LUAD cells transfected with PKM2 siRNA and CHAC1 overexpression lentivirus. CHAC1 lentivirus，PKM2-siRNA and PKM2(WT) or PKM2^I267&268A^(MT) plasmid were reintroduced into A549 cells (C-F). (C, D) Cell motility was determined by Wound-healing assay and transwell migration assays. (E) Using an R package to analyze the correlation between CHAC1 and the expression of GLUT1, ENO1, and LDHA in the TCGA LUAD dataset. (F, G) RT-qPCR and Western blot were used to detect relative mRNA and protein levels of c-Myc. (H) RT-qPCR was used to detect the relative mRNA levels of GLUT1, ENO1 and LDHA. ^***^*p* < 0.001，ns means no statistical significance.

**Figure. S7.**


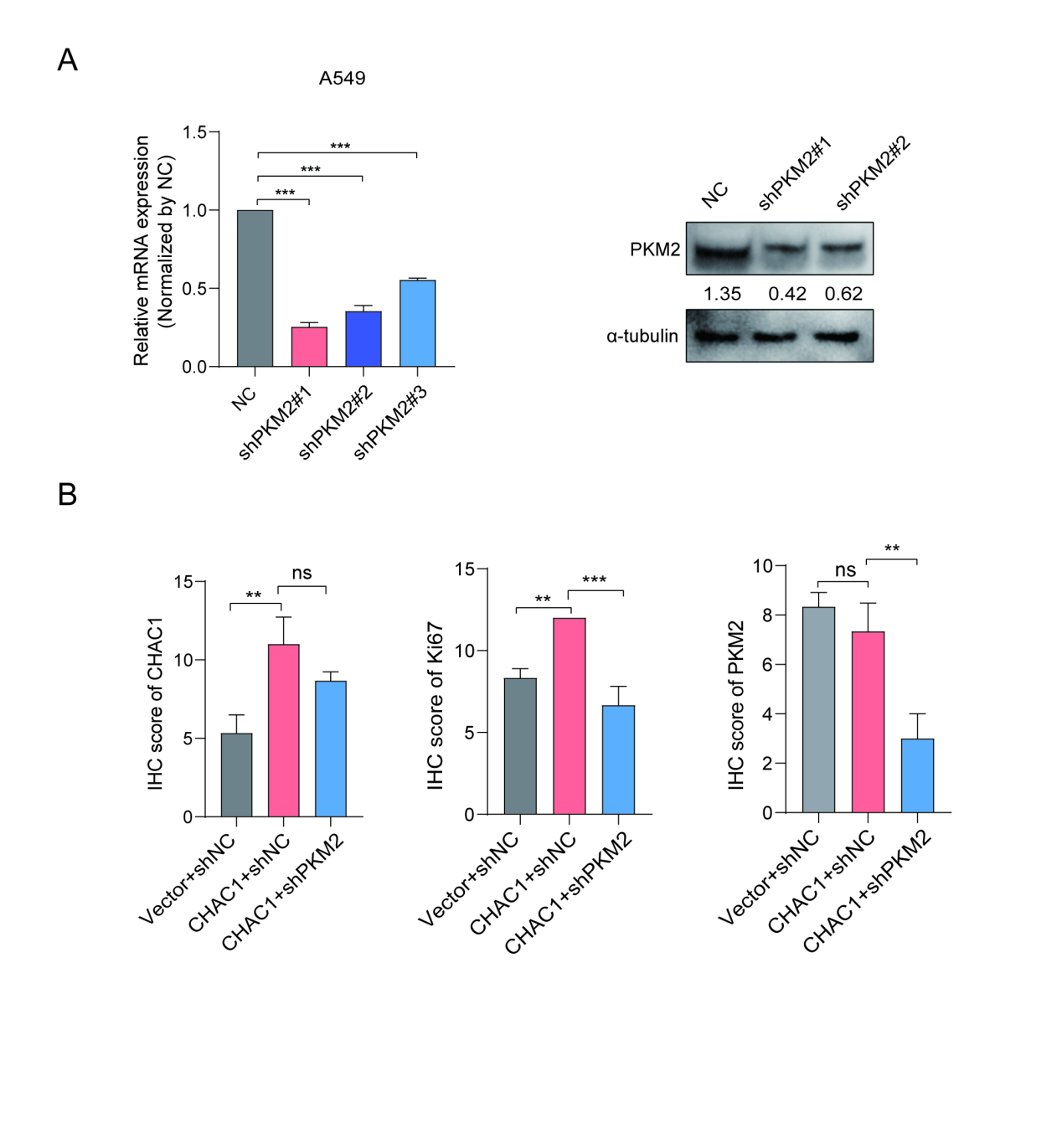
**Supplementary Figure 7.** (A) RT-qPCR and Western blot were used to detect the knockdown efficiency of shPKM2. (B) Immunohistochemical scores of CHAC1, Ki67 and PKM2 in xenograft tumor tissues.

**Supplemental Table 1. Oligonucleotide sequences used in this study.**

| **Oligonucleotide name** | | **Sequence (5' to 3')** |
| --- | --- | --- |
| short hairpin RNAs  (shRNAs) | shRNA-negative control (shNC) | TTCTCCGAACGTGTCACGT |
|  | CHAC1 shRNA#1 | ccACAACCTTGAATACTTGCT |
|  | CHAC1 shRNA#2 | ccTCTTACCCACTTGGTTGTT |
|  | CHAC1 shRNA#3 | gtGACGCTCCTTGAAGATCAT |
|  | PKM2 shRNA#1 | GAAGGGAAAGAACATCAAGAT |
|  | PKM2 shRNA#2 | GTTCGGAGGTTTGATGAAATC |
|  | PKM2 shRNA#3 | CGGGTGAACTTTGCCATGAAT |
| Small interfering RNAs  (siRNAs)  s | E2F1 siRNA#1 | CUACUCAGCCUGGAGCAAGAAdTdT |
|  | E2F1 siRNA#2 | CGCUAUGAGACCUCACUGAAUdTdT |
|  | E2F1 siRNA#3 | CGUGGACUCUUCGGAGAACUUdTdT |
|  | MAZ siRNA#1 | CAGACAAGUGCACUCAACATT |
|  | MAZ siRNA#2 | GAUGCUGAGCUCGGCUUAUAUTT |
|  | MAZ siRNA#3 | CCUUGGAGAAGAAGACAAATT |
|  | UBA2 siRNA#1 | GGAAGAAGAUGCUGAUCAATT |
|  | UBA2 siRNA#2 | GAGUAGAUUUGAUAUCAAATT |
|  | UBA2 siRNA#3 | GAAGAAGAUUCUUCAAAUATT |
|  | PKM2 siRNA#1 | GCUGUGGCUCUAGACACUAAAdTdT |
|  | PKM2 siRNA#2 | GUUCGGAGGUUUGAUGAAAUCdTdT |
|  | PKM2 siRNA#3 | CGUGGAUGAUGGGCUUAUUUCdTdT |
| Primers for qPCR | Human CHAC1 forward | TTTGAGCATCCGTCAGAAGTCC |
|  | Human CHAC1 reverse | CCAGTACAGACACCTGCCCAGT |
|  | Human B-actin forward | TGACGTGGACATCCGCAAAG |
|  | Human B-actin reverse | CTGGAAGGTGGACAGCGAGG |
|  | Human E2F1 forward | CATCCCAGGAGGTCACTTCTG |
|  | Human E2F1 reverse | GACAACAGCGGTTCTTGCTC |
|  | Human MAZ forward | CACGAGGAGAAAGTGCCATGT |
|  | Human MAZ reverse | AGCTCACAGACATGGTGAGGA |
|  | Human HK2 forward | TTGACCAGGAGATTGACATGGG |
|  | Human HK2 reverse | CAACCGCATCAGGACCTCA |
|  | Human PFKP forward | GCATGGGTATCTACGTGGGG |
|  | Human PFKP reverse | CTCTGCGATGTTTGAGCCTC |
|  | Human PKM2 forward | ATAACGCCTACATGGAAAAGTGT |
|  | Human PKM2 reverse | TAAGCCCATCATCCACGTAGA |
|  | Human c-myc forward | CCTACCCTCTCAACGACAGC |
|  | Human c-myc reverse | TTGTTCCTCCTCAGAGTCGC |
|  | Human GLUT1 forward | TCTGGCATCAACGCTGTCTT |
|  | Human GLUT1 reverse | CCGTGTTGACGATACCGGAG |
|  | Human LDHA forward | GATTCAGCCCGATTCCGTTAC |
|  | Human LDHA reverse | AGAGACACCAGCAACATTCATTC |
|  | Human PDK1 forward | ATCACCAGGACAGCCAATACA |
|  | Human PDK1 reverse | CCTCGGTCACTCATCTTCACA |
|  | Human ENO1 forward | TGACCAACCCAAAGAGGATCG |
|  | Human ENO1 reverse | CGATGAGACACCATGACGCC |
| Primers for ChIP | Human CHAC1 promoter E2F1 site 1 forward | CCAGTTTCTCCATTTGCTCACA |
|  | Human CHAC1 promoter E2F1 site 1 reverse | GCGTCGTAAACTTCCGTCTC |
|  | Human CHAC1 promoter E2F1 site 2 forward | GGAAGTTTACGACGCCCAGAG |
|  | Human CHAC1 promoter E2F1 site 2 reverse | CCTTGCCGCTAGTGCATCTG |

**Supplemental Table 2. Primary and second antibodies used in this study.**

| **Antibody** | **Dilutions for WB** | **Dilutions for IHC** | **Dilutions for IF** | | **Dilutions for IP** | **Company** |
| --- | --- | --- | --- | --- | --- | --- |
| CHAC1 | 1:1000 | 1:500 | | 1:200 | 1:100 | Proteintech (15207-1-AP) |
| PKM2 | 1:1000 | 1:500 | | 1:200 | 1:100 | Proteintech (15822-1-AP) |
| SUMO1 | 1:1000 | / | | 1:200 | 1:100 | Abcam（ab32058) |
| UBA2 | 1:1000 | / | | / | 1:100 | Santa Cruz (sc-376305) |
| STAT3 | 1:1000 | / | | / | / | Abcam (ab68153) |
| p-STAT3 (phospho Y705) | 1:1000 | / | | / | / | Abcam (ab76315) |
| E-cadherin | 1:1000 | / | | / | / | Immunoway (YT1454) |
| N-cadherin | 1:1000 | / | | / | / | Proteintech (66219-1-Ig) |
| MMP2 | 1:1000 | / | | / | / | Proteintech (10373-2-AP) |
| MMP9 | 1:1000 | / | | / | / | Abcam (ab76003) |
| α-tubulin | 1:1000 | / | | / | / | Proteintech (11224-1-AP) |
| Lamin B1 | 1:1000 | / | | / | / | Proteintech (66095-1) |
| His | 1:1000 | / | | / | / | CST (#12698) |
| HA | 1:1000 | / | | / | / | CST (#5017) |
| Flag | 1:1000 | / | | / | 1:50 | CST (#14793) |
| E2F1 | 1:1000 | 1：200 | | / | / | Proteintech (66515-1-Ig) |
| Ki-67 | / | 1：200 | | / | / | Abcam (ab15580) |
| GLUT1 | 1:1000 | / | | / | / | Immunoway (YT1928) |
| LDHA | 1:1000 | / | | / | / | Proteintech (19987-1-AP) |
| ENO1 | 1:1000 | / | | / | / | CST (#3810) |
| c-Myc | 1:1000 | / | | / | / | Proteintech (10828-1-AP) |
| Mouse IgG | 1:5000 | / | | / | / | Immunoway (RS0001) |
| Rabbit IgG | 1:5000 | / | | / | / | Immunoway (RS0002) |

**Supplemental Table 3. mRNA expression level of glutathione catabolic process genes in TCGA LUAD samples.**

| **Gene** | **Normal** | **Tumor** | **logFC** | **pValue** |
| --- | --- | --- | --- | --- |
| DPEP1 | 0.412726011 | 1.26008121 | 1.610260447 | 2.67E-13 |
| CHAC2 | 1.444102565 | 3.315019981 | 1.198844355 | 2.46E-17 |
| CHAC1 | 1.363952577 | 2.800434804 | 1.037857358 | 2.61E-12 |
| GGT5 | 4.543840071 | 7.508618014 | 0.724635342 | 7.63E-06 |
| GGT7 | 8.021915304 | 10.54132576 | 0.394037684 | 0.073943242 |
| GGT3P | 0.129756074 | 0.148509686 | 0.194754954 | 0.29264294 |
| GGT2 | 0.40812137 | 0.391916681 | -0.058451275 | 0.001023999 |
| GGT1 | 7.880395094 | 6.881296005 | -0.195587659 | 0.000114374 |
| GGTLC1 | 28.61281419 | 13.68907627 | -1.063636302 | 1.12E-17 |
| GGTLC2 | 0.553151609 | 0.210510888 | -1.393780096 | 3.52E-18 |
| GGTLC3 | 0.303541209 | 0.063416639 | -2.258959071 | 9.64E-19 |

**Supplemental Table 4. Correlation between CHAC1 expression and clinicopathological characteristics of LUAD patients.**

| **Characteristics** | **Tumor CHAC1 expression** | | ***p* values** | **χ^2^** |
| --- | --- | --- | --- | --- |
|  | Low(n=35) | High(n=46) |  |  |
| **Sex**, n (%) |  |  | 0.344 | 0.895 |
| Male | 20 (24.7%) | 31 (38.3%) |  |  |
| Female | 15 (18.5%) | 15 (18.5%) |  |  |
| **Age**, n (%) |  |  | 0.946 | 0.004 |
| ≤ 60 | 18 (22.2%) | 24 (29.6%) |  |  |
| >60 | 17 (21%) | 22 (27.2%) |  |  |
| **Grade**, n (%) |  |  | 0.307 | 2.359 |
| Stage I | 2 (2.5%) | 4 (4.9%) |  |  |
| Stage II | 30 (37%) | 33 (40.7%) |  |  |
| Stage III | 3 (3.7%) | 9 (11.1%) |  |  |
| **T stage**, n (%) |  |  | **0.042** | 4.127 |
| T1 | 10 (12.3%) | 5 (6.2%) |  |  |
| T2-T4 | 25 (30.9%) | 41 (50.6%) |  |  |
| **N stage**, n (%) |  |  | 0.364 | 0.821 |
| N0 | 17 (21%) | 27 (33.3%) |  |  |
| N1-N3 | 18 (22.2%) | 19 (23.5%) |  |  |
| **TNM stage**, n (%) |  |  | 0.803 | 0.061 |
| I-II | 23 (28.4%) | 29 (35.8%) |  |  |
| III-IV | 12 (14.8%) | 17 (21%) |  |  |

**Supplemental Table 5. Univariate and multivariate analysis of factors associated with survival in LUAD.**

| **Characteristics** | **Total (N)** | **Univariate analysis** | | **Multivariate analysis** | |
| --- | --- | --- | --- | --- | --- |
|  |  | HR (95% CI) | *p* values | HR (95% CI) | *p* values |
| **Sex** | 81 |  |  |  |  |
| Male | 51 | Reference |  |  |  |
| Female | 30 | 0.675 (0.374 - 1.218) | 0.192 |  |  |
| **Age** | 81 |  |  |  |  |
| ≤ 60 | 42 | Reference |  |  |  |
| >60 | 39 | 0.918 (0.531 - 1.585) | 0.758 |  |  |
| **Grade** | 81 |  |  |  |  |
| Stage II | 63 | Reference |  |  |  |
| Stage III | 12 | 0.648 (0.275 - 1.527) | 0.321 |  |  |
| Stage I | 6 | 1.309 (0.517 - 3.315) | 0.570 |  |  |
| **T stage** | 81 |  |  |  |  |
| T2-T4 | 66 | Reference |  | Reference |  |
| T1 | 15 | 0.309 (0.122 - 0.780) | 0.013 | 0.402 (0.150 - 1.077) | **0.070** |
| **N stage** | 81 |  |  |  |  |
| N1-N3 | 37 | Reference |  | Reference |  |
| N0 | 44 | 0.617 (0.356 - 1.070) | 0.086 | 1.326 (0.553 - 3.179) | 0.527 |
| **TNM stage** | 81 |  |  |  |  |
| I-II | 52 | Reference |  | Reference |  |
| III-IV | 29 | 2.351 (1.327 - 4.166) | 0.003 | 2.656 (1.118 - 6.311) | **0.027** |
| **CHAC1 (High vs. Low)** | 81 | 1.095 (1.014 - 1.183) | 0.021 | 1.087 (1.002 - 1.179) | **0.044** |

**Supplemental Table 8. JASPAR predicts transcription factor binding CHAC1 promoter scores**

| **Name** | **Score** | **Sequence ID** | **Start** | **End** | **Predicted sequence** |
| --- | --- | --- | --- | --- | --- |
| YY1 | 14.241599 | NC_000015.10:40951471-40953570 | 827 | 838 | CAACATGGCTTC |
| MAZ | 13.436413 | NC_000015.10:40951471-40953570 | 1478 | 1488 | GGCCCCTCCTG |
| E2F1 | 10.443947 | NC_000015.10:40951471-40953570 | 1458 | 1465 | TTTCCCGC |
| E2F1 | 10.277139 | NC_000015.10:40951471-40953570 | 1598 | 1608 | CTAGCGGCAAG |
| STAT1 | 10.241949 | NC_000015.10:40951471-40953570 | 393 | 403 | CTTCTAAGAAG |
| YY1 | 8.38313 | NC_000015.10:40951471-40953570 | 855 | 860 | GCCATC |
| JUND | 8.2631445 | NC_000015.10:40951471-40953570 | 1087 | 1097 | AATGATTAATC |
