# Supplementary material for: CHAC1 blockade suppresses progression of lung adenocarcinoma by interfering with glucose metabolism via hijacking PKM2 nuclear translocation
Source: Cell Death Dis. 2024 Oct 5;15(10):728. doi: 10.1038/s41419-024-07114-6 (PMC11455913; doi:10.1038/s41419-024-07114-6)

Fig 1I

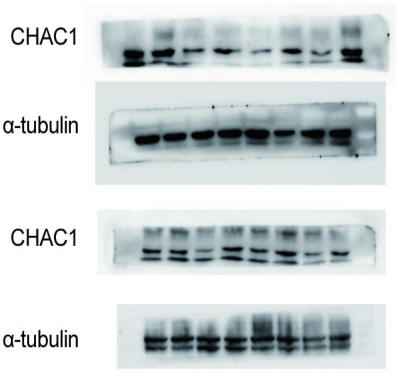


Fig 1J

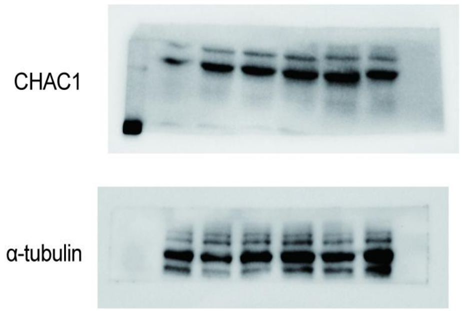


Fig 2A

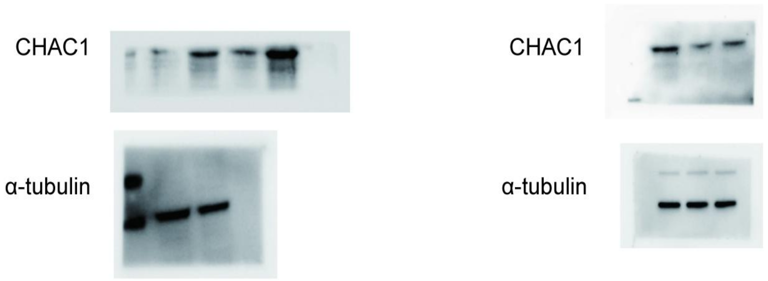


Fig 2I

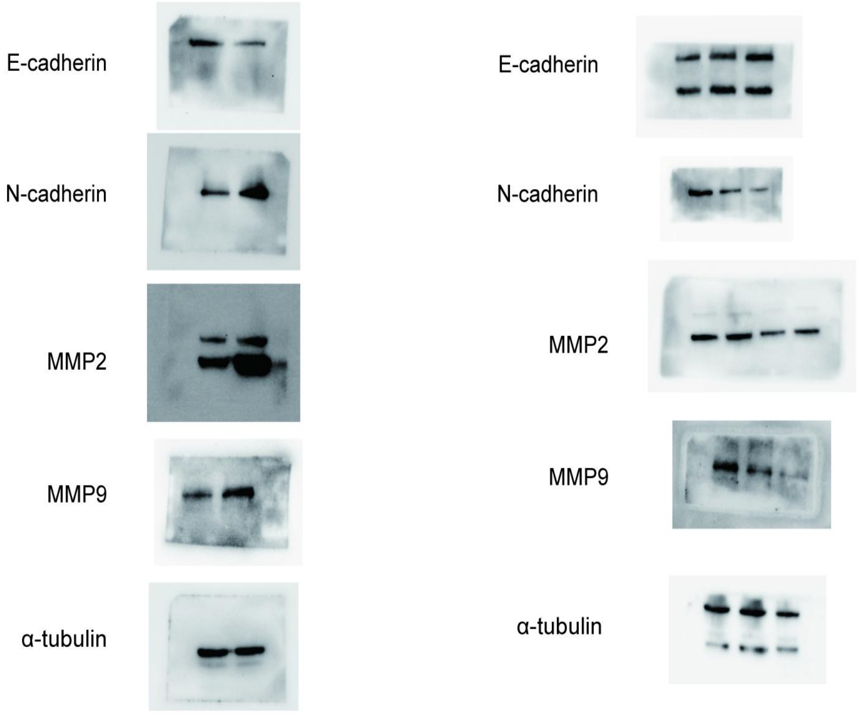

Fig 4C

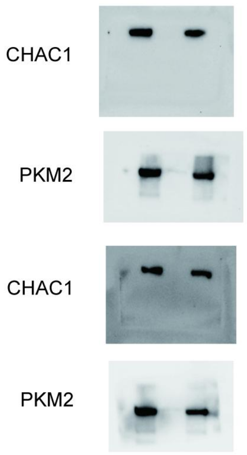


Fig 4D

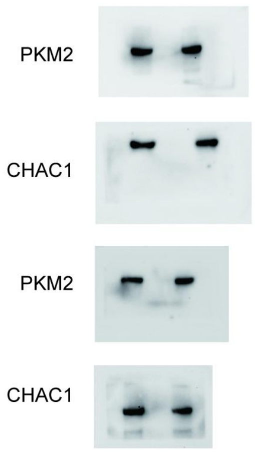


Fig 4E

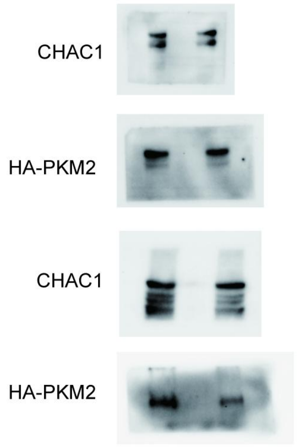


Fig 4G

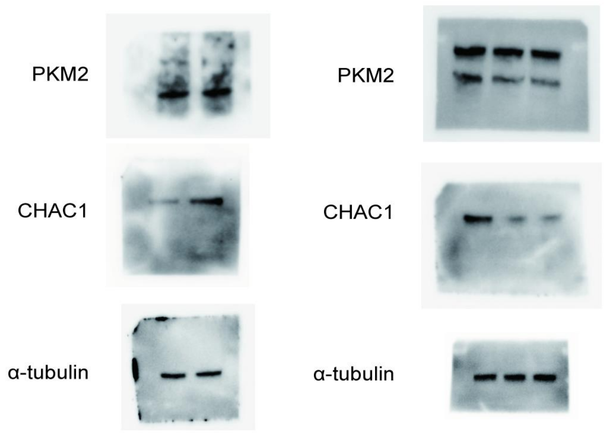


Fig 4H

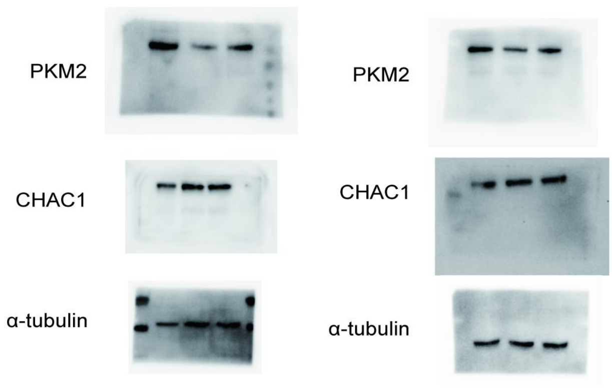

Fig 5B

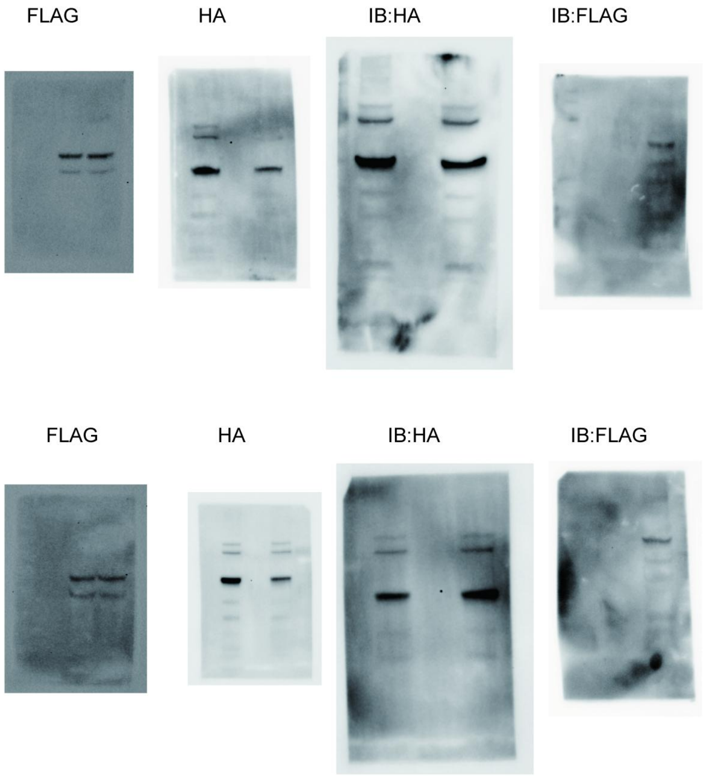


Fig 5C

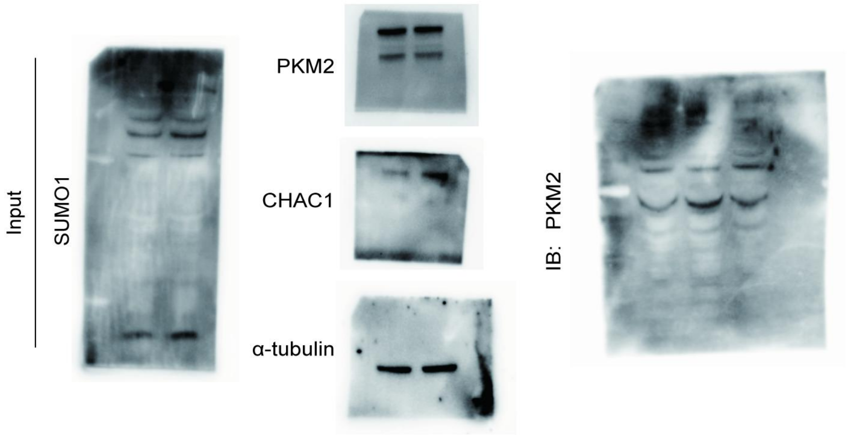


Fig 5D

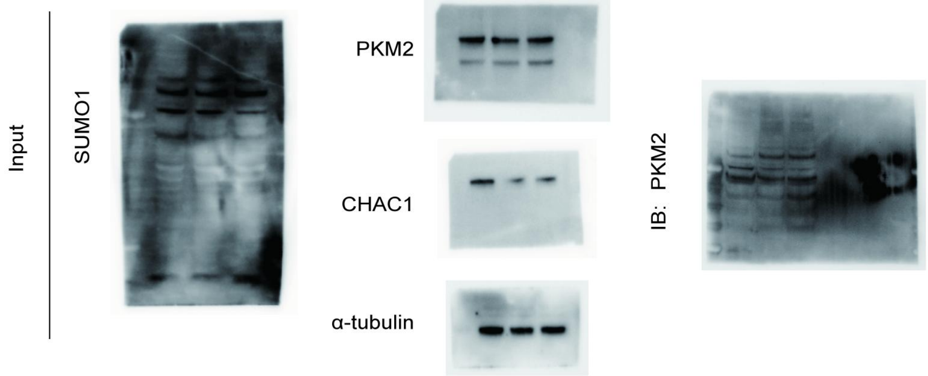

Fig 5I

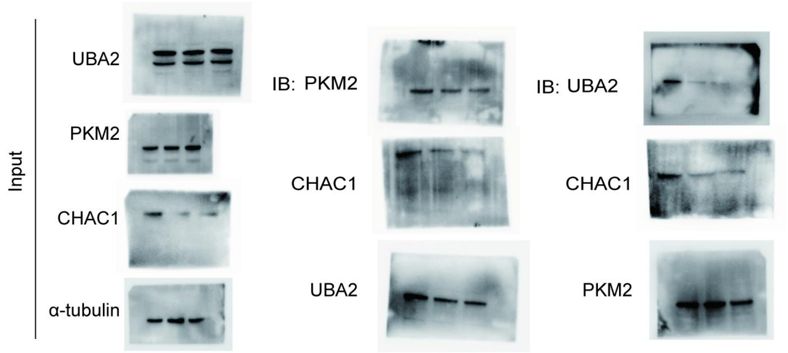


Fig 5J

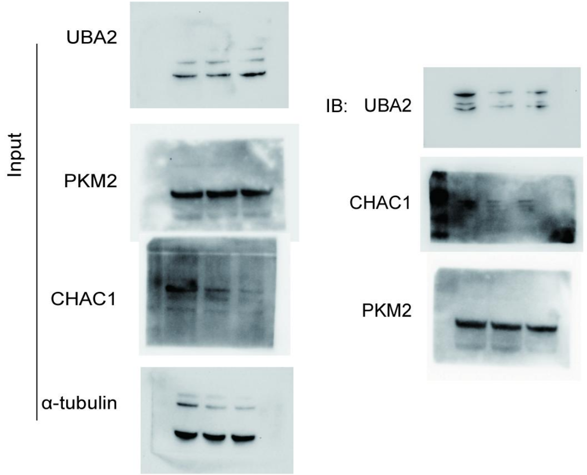


Fig 6A

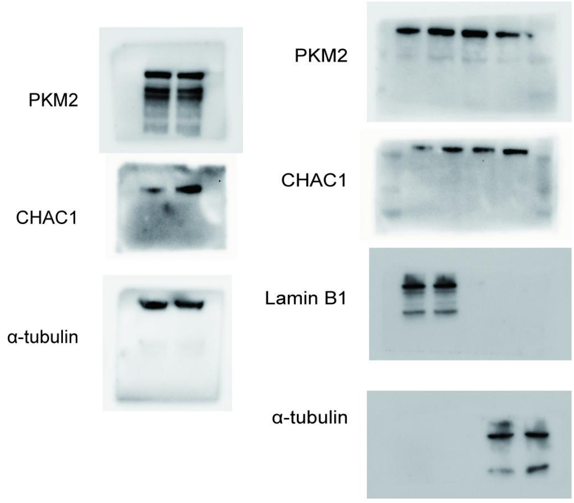


Fig 6C

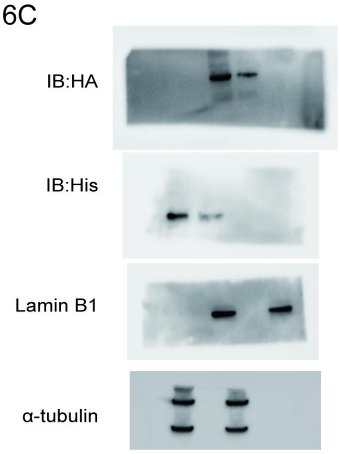

Fig 5E

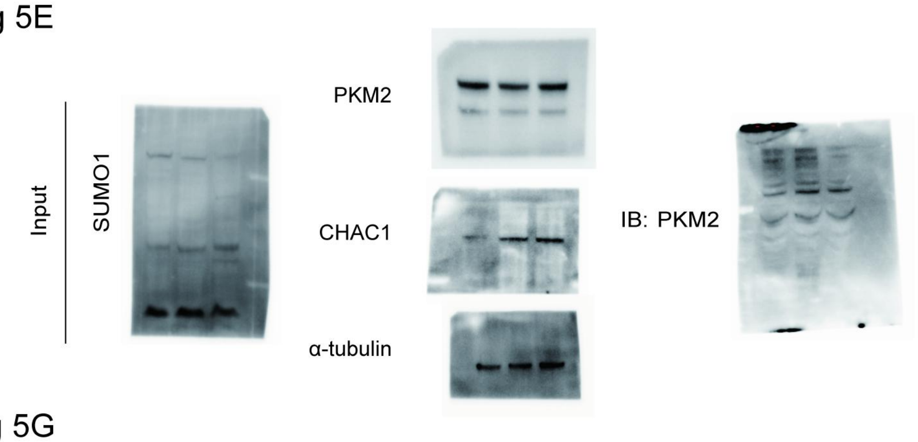


Fig 5G

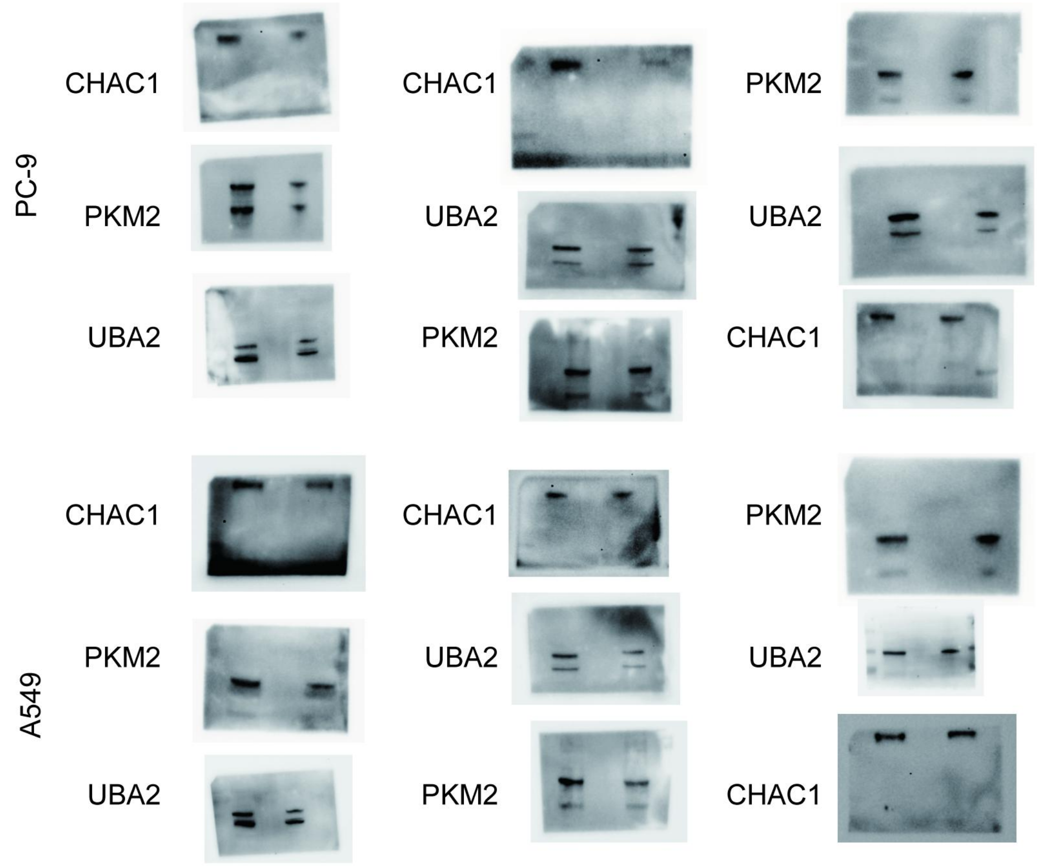


Fig 5H

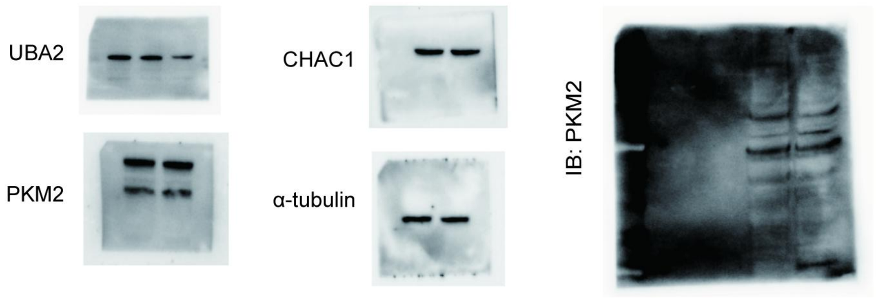

Fig 6H

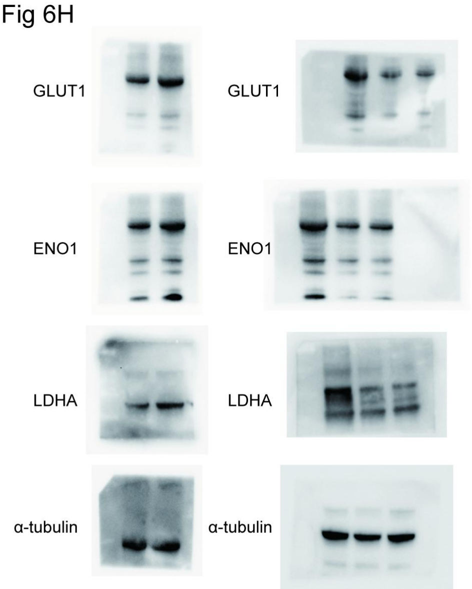


Fig 6J

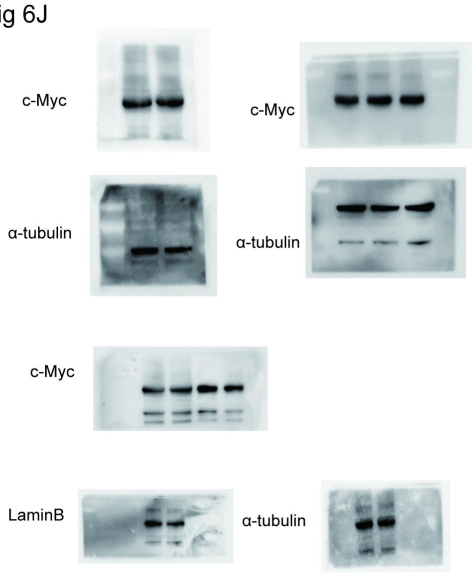


Fig 6K

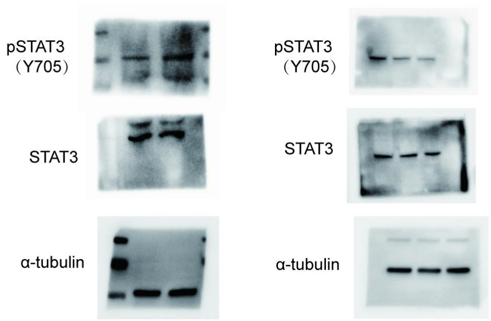


Fig 6J

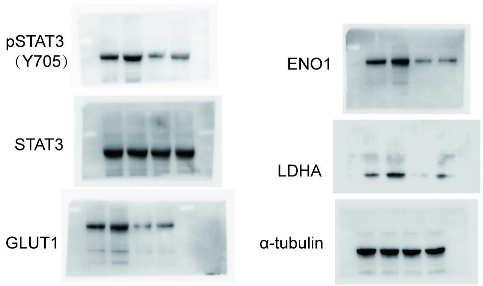


Fig 6M

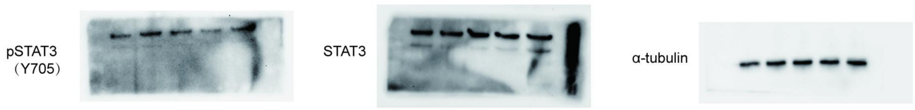


Fig 7G

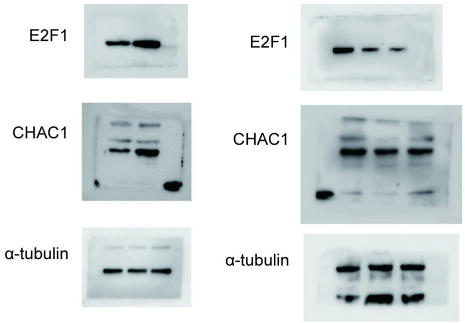


Fig S5

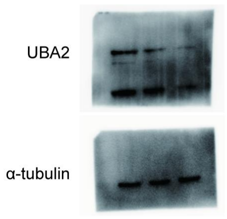


Fig S7

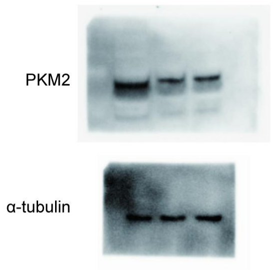

Supplement: Supplementary file 2 — Supplementary Data [file 41419_2024_7114_MOESM2_ESM.pdf]
